# Lens-free on-chip 3D microscopy based on wavelength-scanning Fourier ptychographic diffraction tomography

**DOI:** 10.1038/s41377-024-01568-1

**Published:** 2024-09-05

**Authors:** Xuejuan Wu, Ning Zhou, Yang Chen, Jiasong Sun, Linpeng Lu, Qian Chen, Chao Zuo

**Affiliations:** 1 Smart Computational Imaging (SCI) Laboratory, Nanjing University of Science and Technology, No. 200 Xiaolingwei Street, 210094 Nanjing, Jiangsu China; 2 Smart Computational Imaging Research Institute (SCIRI) of Nanjing University of Science and Technology, 210094 Nanjing, Jiangsu China; 3 Jiangsu Key Laboratory of Spectral Imaging & Intelligent Sense, No. 200 Xiaolingwei Street, 210094 Nanjing, Jiangsu China

**Keywords:** Imaging and sensing, Transmission light microscopy

## Abstract

Lens-free on-chip microscopy is a powerful and promising high-throughput computational microscopy technique due to its unique advantage of creating high-resolution images across the full field-of-view (FOV) of the imaging sensor. Nevertheless, most current lens-free microscopy methods have been designed for imaging only two-dimensional thin samples. Lens-free on-chip tomography (LFOCT) with a uniform resolution across the entire FOV and at a subpixel level remains a critical challenge. In this paper, we demonstrated a new LFOCT technique and associated imaging platform based on wavelength scanning Fourier ptychographic diffraction tomography (wsFPDT). Instead of using angularly-variable illuminations, in wsFPDT, the sample is illuminated by on-axis wavelength-variable illuminations, ranging from 430 to 1200 nm. The corresponding under-sampled diffraction patterns are recorded, and then an iterative ptychographic reconstruction procedure is applied to fill the spectrum of the three-dimensional (3D) scattering potential to recover the sample’s 3D refractive index (RI) distribution. The wavelength-scanning scheme not only eliminates the need for mechanical motion during image acquisition and precise registration of the raw images but secures a quasi-uniform, pixel-super-resolved imaging resolution across the entire imaging FOV. With wsFPDT, we demonstrate the high-throughput, billion-voxel 3D tomographic imaging results with a half-pitch lateral resolution of 775 nm and an axial resolution of 5.43 μm across a large FOV of 29.85 mm^2^ and an imaging depth of >200 μm. The effectiveness of the proposed method was demonstrated by imaging various types of samples, including micro-polystyrene beads, diatoms, and mouse mononuclear macrophage cells. The unique capability to reveal quantitative morphological properties, such as area, volume, and sphericity index of single cell over large cell populations makes wsFPDT a powerful quantitative and label-free tool for high-throughput biological applications.

## Abstract (web summary)

We present wavelength-scanning Fourier ptychographic diffraction tomography (wsFPDT), a lens-free on-chip tomography method for high-throughput, motion-free, pixel-super-resolved 3D imaging with quasi-uniform resolution across the sensor’s full field of view.

## Graphical abstract

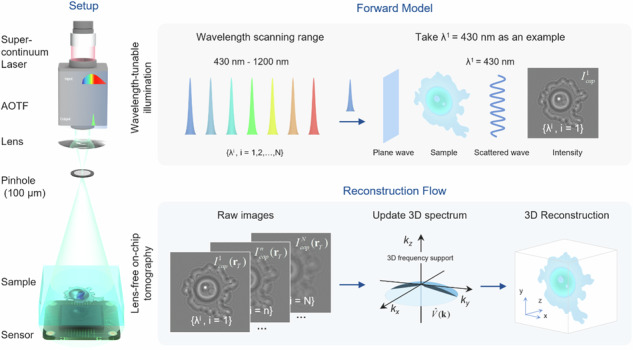

## Introduction

High-throughput microscopy, i.e., recording large field-of-view (FOV) images without compromising spatial and temporal resolution, is of critical importance to imaging science^1,2^. For example, in stem cell biology^3^, cancer diagnosis^4^, and drug discovery^5^, many biomedical applications require the identification and isolation of specific cell types from a heterogeneous cell population. However, these events are often rare, and we often need high-content quantitative analysis of multiple events in a large population of cells^6–9^. Therefore, the ideal microscopic imaging technology for these purposes should be able to image and analyze tens of thousands of cells at once over a large FOV. However, for a conventional microscope, the acquired information is limited by the spatial-bandwidth product (SBP) of the imaging system, which is typically on the order of 10 megapixels^10,11^. More specifically, conventional microscopes suffer from the trade-off between resolution and FOV due to the constraints of the Lagrange invariance of objective lenses. Low-magnification objective lenses offer a large FOV at the expense of resolution, while high-magnification objective lenses provide a high resolution at the cost of the FOV. Instead of mechanically scanning the microscope’s FOV and stitching overlapping images, recently developed computational microscopy techniques present novel prospects for generating high-resolution wide-field images with alternative elegant solutions, such as synthetic aperture holography^12–16^, Fourier ptychography microscopy (FPM)^17–20^, and lens-free on-chip microscopy (LFOCM)^21–23^. Among these, LFOCM is probably the most promising technique in regard to high-throughput imaging thanks to its unique advantages of achieving a large effective numerical aperture (NA) approaching 1 across the native FOV of the imaging sensor without requiring any lenses and other intermediate optical components^24,25^. This further simplifies the imaging setup while effectively circumvents the optical aberrations and chromatism inherent in conventional lens-based imaging systems. Furthermore, the entire system can be built in a miniaturized and cost-effective manner, offering a potential and promising solution to point-of-care diagnostics in resource-limited environments^26,27^.

Despite all the advantages, LFOCM still faces several technical challenges in its practical application. Firstly, because the sample is placed close to the surface of the image sensor (without geometric magnification), image resolution is limited by the pixel size of the sensor pixel (typically >1 μm), which is well below its theoretical optical diffraction limit. To overcome this limitation, many pixel-super-resolution (PSR) methods have been proposed to reduce the effective pixel size, such as sub-pixel shifting of illumination sources^28^, sub-pixel lateral translation of image sensors^29^, active parallel plates scanning^30^, wavelength scanning^31,32^, axial scanning of sample-to-sensor distances^33,34^, and mask-modulation-based Wirtinger flow^35,36^. These methods surpass the Nyquist-Shannon sampling resolution limitation imposed by the sensor pixel size, making high-resolution lens-free microscopy imaging possible^37^. Secondly, quantitative phase imaging (QPI) can visualize and quantify the optical thickness variation of unlabeled transparent biological samples without the need for specific staining or exogenous contrast agents^38,39^. However, LFOCM was initially designed for imaging absorptive or stained samples, recording the quasi-in-focus shadows of the sample directly without the need for any image reconstruction procedures^21,40^. Nevertheless, when the sample-to-sensor distance is large, and the source coherence is sufficient to produce an apparent diffraction (or equivalently interference) effect, one can reconstruct a complex amplitude image (including both amplitude and phase) of the sample through phase retrieval and digital refocusing^25,34,41^. Therefore, LFOCM can also be adopted as a high-throughput QPI method that is well suited for time-lapse imaging unstained live cells or samples that are non-fluorescent or cannot be fluorescently tagged^32,42,43^. Finally, most current LFOCM techniques are only designed for two-dimensional (2D) imaging of thin samples. However, in practice, many biological cells and tissues will not often be thin, leading to the issue that the three-dimensional (3D) morphological parameters of samples cannot be obtained precisely^44^. For imaging such thick 3D samples that cannot be simply modeled as a 2D complex transmittance function, optical diffraction tomography (ODT) techniques are preferable to reconstruct a “true 3D” image so that the volumetric refractive index (RI) information inside the sample can be accessible^45–48^.

Nevertheless, until now, only a few works have explicitly considered the challenging issue of ***lens-free on-chip tomography*** (LFOCT). The standard techniques for ODT are achieved by combining QPI with tomography techniques^49–51^. Isikman et al.^52^ demonstrated an LFOCT platform by rotating the illumination arm. The diffraction patterns of the sample at various illumination angles were recorded and reconstructed, and the standard computed tomography algorithms (filtered back projection/inverse Radon transform) were used to synthesize a 3D tomogram. However, since the diffraction effect of the sample is ignored in their model, only absorptive samples can be imaged. In addition, mechanical beam rotation using robotic arms makes the data acquisition process complicated and time-consuming. Zuo et al.^53^ proposed a compact LFOCT platform that used a programmable color LED matrix to illuminate the sample at multiple wavelengths and angles without any time-consuming mechanical operations, and reconstructed the wide-field volumetric RI distribution of thick specimens based on the multi-wavelength phase retrieval and the diffraction tomography theory that explicitly accounted for the diffraction effect. Berdeu et al.^54^ used a 360° axially rotatable robotic arm equipped with a light source at a fixed tilt angle of 45° to build an LFOCT platform. Taking into account the diffraction effect, they calculated the complex amplitude at each illumination angle by the phase ramp or 2D phase retrieval method. Then the full 3D reconstructions were obtained based on the Fourier diffraction theorem. Luo et al.^55^ built a scan-free multi-angle illumination LFOCT system based on only four laser diodes (LDs) and applied a fast proximal gradient algorithm with composite regularization to perform tomographic imaging using only 4 images captured under different illumination directions.

Although the above-mentioned methods have demonstrated the feasibility of 3D tomographic imaging on an LFOCM platform at the proof-of-concept level, they generally rely on multi-angle illuminations based on mechanical beam scanning or fixed multi-source schemes (LED arrays or LDs). Particularly, the introduction of mechanical scanning devices leads to a complex experimental configuration. Moreover, as the sample can only be imaged in the defocused plane, tilted illumination changes the beam propagation direction after passing through the sample, resulting in a series of shifted and distorted diffraction patterns, whose relative translations are proportional to the illumination angle and sample-to-sensor distance^52,53^. These displaced image datasets not only make the image reconstruction more complicated (additional image registration and rectification are needed for a precise and high-resolution reconstruction) but also significantly reduce the imaging resolution for samples located at the edge of the image sensor compared with the central region (due to ‘flying-out’ data loss at large-angle illumination)^52,53^. Therefore, high-throughput motion-free LFOCT with a quasi-uniform, pixel-super-resolved imaging resolution across the whole image FOV is still an open quest.

In this paper, for the first time to our knowledge, we propose a wavelength-scanning scheme for LFOCT, termed wavelength-scanning Fourier ptychographic diffraction tomography (wsFPDT), to achieve high-throughput, motion-free, label-free 3D tomography with a quasi-uniform imaging resolution across the full FOV of the image sensor. Instead of using angularly-variable illuminations as in the state-of-the-arts, in wsFPDT, the sample is sequentially scanned by on-axis illumination with variable wavelengths spanning from 430 to 1200 nm. The corresponding under-sampled diffraction patterns are processed based on an iterative ptychographic reconstruction algorithm to recover the pixel-super-resolved 3D RI distribution of the sample. The on-axis wavelength-scanning scheme not only eliminates the need for mechanical motion during image acquisition but offers the quasi-uniform imaging resolution across the entire FOV by addressing all the challenges, including image shift, distortion, and sub-pixel registration, originating from multi-angle illuminations. With wsFPDT, we demonstrate the high-throughput, billion-voxel 3D tomographic imaging results with a half-pitch lateral resolution of 775 nm and an axial resolution of 5.43 μm across a large FOV of 29.85 mm^2^ and an imaging depth of over 200 μm. The effectiveness of the proposed wsFPDT method is experimentally validated by imaging resolution targets, micro-polystyrene beads, and various biological specimens. Offering a decent spatial resolution and quantitative 3D RI imaging capability across a large imaging volume, wsFPDT could in general benefit various high-throughput biological and biomedical applications.

## Results

### Optical setup

**Fig. 1:**
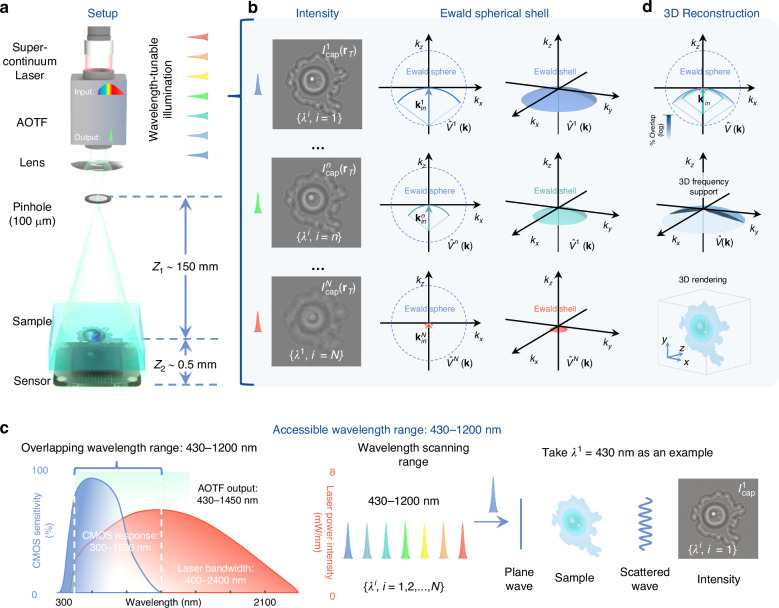
**Experimental schematic of the wavelength-scanning LFOCT platform**. **a** Diagram of the optical setup. **b** The frequency support of the sample for each intensity image is a 2D Ewald spherical shell. **c** Schematic diagram of the experimental procedure. **d** The Ewald shells corresponding to the accessible wavelengths fill the frequency space sequentially, constituting the frequency support domain of our LFOCT platform. The 3D spectrum is updated with the use of the wsFPDT method, and the 3D RI distribution of the sample can be obtained after the inverse Fourier transform

The schematic of our wavelength-scanning LFOCT platform is depicted in Fig. 1a. The illumination from a supercontinuum laser source (YSL, SC-Pro7) with a broad spectral range from 400 to 2400 nm is filtered by an acoustic-optical tunable filter (AOTF, YSL AOTF-Pro, bandwidths: 2–11 nm, RF1: 430–780 nm, RF2: 780–1450 nm, 1 nm interval). The output quasi-monochromatic illumination is then spatially filtered by a 100 μm pinhole to create an ideal spherical wavefront for sample illumination. After the spherical wavefront propagates a relatively long distance in the air (*Z*_1_ distance ~150 mm), the resulting quasi-plane wave interacts with the sample of interest and generates in-line diffraction patterns, which are recorded by a board-level monochrome CMOS sensor (1.67 μm, 3872 × 2764, The Imaging Source, DMK 24UJ003) positioned close to the sample (*Z*_2_ distance <1 mm). The response time of AOTF, being only a few tens of microseconds, is significantly faster than mechanical scanning by several orders of magnitude^52,56,57^. As a result, it can greatly enhance the efficiency of data acquisition. Subsection “Imaging acquisition and analysis” will provide details on the image acquisition process, such as the experimental control, the number of images, and the time required.

### Non-interferometric ODT with wavelength scanning

The wsFPDT method is a non-interferometric ODT technique that solves the inverse scattering problem and reconstructs the scattering potential of a thick 3D sample using only intensity measurements. However, instead of using angularly-variable illuminations as in conventional ODT methods, wsFPDT employs only on-axis quasi-monochromatic plane wave illumination but varies the wavelength to gradually extend the accessible object frequencies in the 3D Fourier domain. As detailed in Supplementary Note S1, the Fourier diffraction theorem suggests that under first-order Born or Rytov approximation, the 2D scattered field information under on-axis quasi-monochromatic illumination (that can be measured or retrieved) only occupies a semi-spherical cap in the 3D Fourier space, which is a subsection of the Ewald sphere with a radius of ${k}_{m}^{i}={n}_{m}\frac{2\pi }{{\lambda }^{i}}$, where *n*_*m*_ is the RI of the surrounding medium and *λ*^*i*^ is the illumination wavelength. Due to the wavelength-dependent nature of the Ewald sphere, illuminating the object sequentially with different wavelengths will change the radius of the semi-spherical cap, enlarging the accessible object frequencies in the 3D Fourier domain.

For ODT techniques, the lateral and axial imaging resolution can be theoretically predicted by the support for accessible object frequencies in the 3D Fourier domain. Consequently, the wavelength-scanning range of our LFOCT platform is crucial to the imaging performance of the wsFPDT method. It is obvious that a wider wavelength scanning range provides better imaging resolution and depth sectioning capability. Nevertheless, considering the spectral coverage of the light source and the spectral response range of the CMOS sensor, the maximum possible wavelength scanning range used in the LFOCT platform is from 430 to 1200 nm, as illustrated in Fig. 1c. When the illumination wavelength is tuned from 430 to 1200 nm, the radius of the Ewald spherical shell gradually shrinks and achieves a certain spectral coverage of the object spectrum eventually. Theoretically, the lower limit for the scanning wavelength (shortest wavelength) determines the maximum frequency extension and thus the lateral and axial imaging resolution. However, the actual imaging performance is often compromised due to the relatively limited angular response and large pixel size of the image sensor^23,58^. Additional computational algorithms involving pixel super-resolution are required to overcome the sampling limitation and recover more high-frequency information from the aliased low-resolution images (detailed in Subsection “The wsFPDT reconstruction algorithm”). On the other hand, the upper limit for the scanning wavelength (longest wavelength) determines how well the low-frequency region, the so-called ‘missing cone’ along the *z*-axis can be filled. Although increasing the upper limit of the wavelength range can provide better recovery of the low-frequency component (see the comparative simulation Fig. S4 in Supplementary Note S4 for more details), it is impractical in our system due to the limited spectrum range of the CMOS sensor. Thus, numerical postprocessing based on hybrid non-negativity constraint and total variation (TV) regularization is employed in our wsFPDT method to alleviate the low-frequency missing and RI under-estimation originating from the ‘missing cone’ problem (detailed in Subsection “The wsFPDT reconstruction algorithm” and Supplementary Note S2).

### The wsFPDT reconstruction algorithm

As mentioned earlier, as a non-interferometric ODT technique, the wsFPDT method needs to address the inverse scattering, synthetic aperture, and aliasing problems simultaneously through intensity-only measurements. Following the success of the FPDT method in lens-based ODT applications^59,60^, we extended it to lens-free imaging to derive the relation to establish an accurate image formation model linking the sample scalar potential to the measured data (defocused and aliased low-resolution intensity image at different illumination wavelengths).

**Fig. 2:**
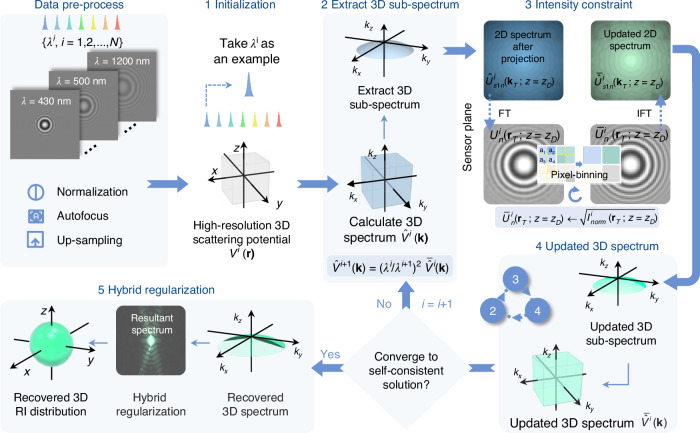
**Flowchart of the reconstruction algorithm for the proposed wsFPDT method**. The whole process of wsFPDT is summarized as: Step 1: initialize the scattering potential *V*^*i*^(**r**); Step 2: obtain the scattering potential’s spectrum ${\hat{V}}^{i}({\bf{k}})$ and extract 3D sub-spectrum; Step 3: obtain the complex amplitude ${U}_{n}^{i}({\bf{r}})$ at the sensor plane through projection and propagation, implement intensity constraints, and transform it to the frequency domain to obtain the updated complex amplitude spectrum ${\bar{\hat{U}}}_{s1n}^{i}({{\bf{k}}}_{T};z={z}_{D})$; Step 4: remap ${\bar{\hat{U}}}_{s1n}^{i}({{\bf{k}}}_{T};z={z}_{D})$ to the 3D Ewald shell and insert it into the corresponding position to update the 3D scattering potential spectrum ${\bar{\hat{V}}}^{i}({\bf{k}})$, then convert to the next wavelength; Steps 2–4 constitute the sub-iteration of the update procedure; Step 5: use a hybrid regularization method to further fill the spectrum and obtain the recovered RI distribution of the sample

Figure 2 illustrates the data processing flowchart of the wsFPDT algorithm for 3D RI reconstruction, taking a simulated microsphere as an example. Firstly, the experimental data must undergo preprocessing, including normalizing image intensity, determining the defocus distance *z*_*D*_ using the autofocus algorithm^61,62^, and configuring an upsampling factor. Subsequently, the iterative reconstruction procedure entails three primary components: (i) making an initial guess of the high-resolution *k*-space scattering potential, (ii) iteratively updating the 3D scattering potential spectrum based on spatial intensity constraints, and (iii) implementing a hybrid regularization to complete the missing information.

- **Initialization of 3D scattering potential spectrum**

Since our previous work has shown that the initial value is not critical to the final reconstruction result^59,63^, we use the RI values of the surrounding medium to fill the sample space, and then perform a Fourier transform to obtain the 3D scattering potential spectrum.

- **3D scattering potential spectrum update**

This iterative process is performed alternatively between the spatial and the Fourier domains, following these sub-steps:

1. Extract 3D sub-spectrum. We acquire the 3D Ewald spectrum at an arbitrary illumination wavelength *λ*^*i*^ from the 3D scattering potential spectrum ${\hat{V}}^{i}({\bf{k}})$. The 3D Ewald spectrum is projected onto a 2D plane and converted into a 2D scattering field. The initialized first-order scattering field ${\hat{U}}_{s1n}^{i}({{\bf{k}}}_{T};z={z}_{D})$ can be related to the scattering potential in 3D Fourier space according to the Fourier diffraction theorem^45^:
  1 $$\begin{array}{rcl}&&{\hat{U}}_{s1n}^{i}({{\bf{k}}}_{T};z={z}_{D})=-\frac{j}{4\pi {k}_{z}}\exp (2\pi j{k}_{z}{z}_{D})\\ &&{\hat{V}}^{i}\left({\bf{k}}-{{\bf{k}}}_{in}^{i}\right)\delta \left({k}_{z}-\sqrt{{{k}_{m}^{i}}^{2}-{\left\vert {{\bf{k}}}_{T}\right\vert }^{2}}\right)\end{array}$$
2. Impose intensity constraint. Under Rytov approximation, the relationship between the initialized first-order scattered field and the measured intensity can be established as
  2 $$\begin{array}{ll}{I}_{n}^{i}({{\bf{r}}}_{T};z={z}_{D})\,=\,{\left\vert {U}_{n}^{i}({{\bf{r}}}_{T};z = {z}_{D})\right\vert }^{2}\\\qquad\qquad\qquad\,\,\,\,=\,{\left\vert \exp [{U}_{s1n}^{i}({{\bf{r}}}_{T};z = {z}_{D})]\right\vert }^{2}\end{array}$$
  The low-resolution intensity image is used as an amplitude constraint to update the measured intensity to obtain ${I}_{change}^{i}({{\bf{r}}}_{T};z={z}_{D})$. Then the updated first-order scattered field is obtained with a relaxation factor *α*:
  3 $$\begin{array}{ll}{\bar{U}}_{s1n}^{i}({{\bf{r}}}_{T};z={z}_{D})\,=\,\ln \left(\alpha \sqrt{{I}_{change}^{i}({{\bf{r}}}_{T};z={z}_{D})}\right.\\\qquad\qquad\qquad\qquad\exp \left\{j\arg \left[{U}_{n}^{i}({{\bf{r}}}_{T};z={z}_{D})\right]\right\}\\\qquad\qquad\qquad\qquad+\left.(1-\alpha ){U}_{n}^{i}({{\bf{r}}}_{T};z={z}_{D})\right)\end{array}$$
3. Update scattering potential spectrum. The updated 2D sub-spectrum ${\bar{\hat{U}}}_{s1n}^{i}({{\bf{k}}}_{T};z={z}_{D})$ is remapped to the 3D Ewald spherical shell and inserted into the corresponding position in the frequency space, completing an iteration for updating the 3D scattering potential spectrum.

After the updated scattering potential spectrum is converted to the initial value at the next wavelength, ${\hat{V}}^{i+1}({\bf{k}})={({\lambda }^{i}/{\lambda }^{i+1})}^{2}{\bar{\hat{V}}}^{i}({\bf{k}})$, the iteration process continues to repeat the three sub-steps above (corresponding to Steps 2–4 in Fig. 2). The process is repeated for all illumination wavelengths until the wsFPDT converges within all intensity images.

- **Hybrid regularization**

Due to the limited ranges of illumination angle and wavelength scanning achievable by our experimental configuration, it is still not possible to recover the region close to the origin of the frequency space. To address this issue, in the final step of the reconstruction algorithm, we use a hybrid regularization method^14^ that combines TV regularization with non-negativity constraint to fill this missing information computationally. After applying a 3D inverse Fourier transform, we can reconstruct the scattering potential of the object and obtain the corresponding 3D volumetric RI distribution. For a detailed description of the working flow of wsFPDT for 3D RI reconstruction, please refer to Supplementary Note S2. Additionally, Supplementary Note S3 provides simulation and experimental results of microspheres, which confirm the effectiveness of wsFPDT in reconstructing the RI of label-free 3D samples.

### Tomographic RI reconstruction of the tilted phase resolution target

**Fig. 3:**
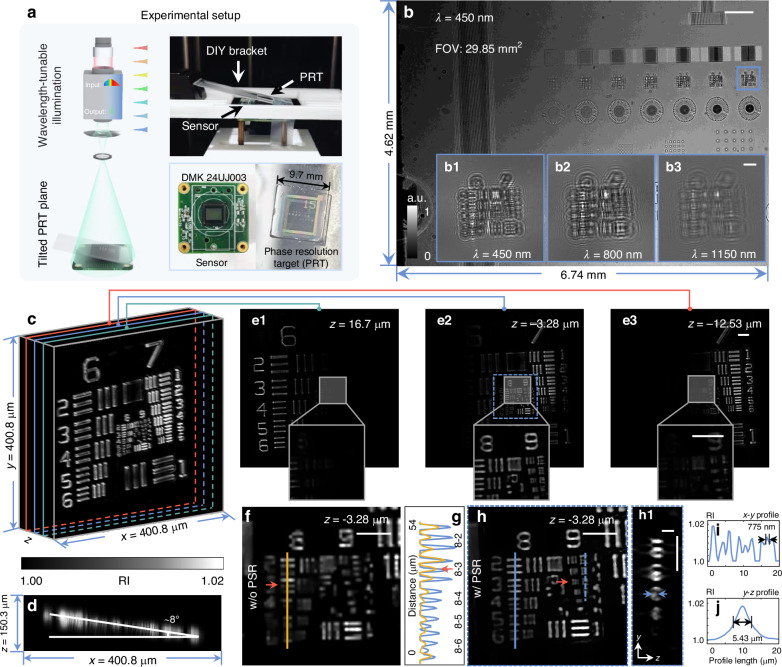
**3D reconstruction of a tilted phase resolution target**. **a** Diagram of the experimental setup. **b** Full-FOV intensity image of the PRT collected by the sensor, and the blue box representing the target area selected for 3D recovery. The insets show the diffraction patterns of the target area at different illumination wavelengths. **c** Volume rendered image of the tilted PRT reconstructed by the wsFPDT method. **d** *x* − *z* projection of RI reconstruction illustrating the tilt angle of ~ 8°. **e1**–**e3** The cross-sections of the corresponding RI distribution for three different axial planes. **f** Reconstruction of Groups 8–9 obtained without the PSR algorithm during the iteration. **g** The line profiles across the Elements 2–6 of Group 8 in **f** and **h**. **h** Reconstruction of Groups 8–9 elements with the PSR algorithm (enlarged view corresponding to the dashed box in **e2**). **h1** The *y* − *z* slice corresponding to the dashed line in **h**. **i** The line profile corresponding to the Elements 1–3 of Group 9 in **h**. **j** The axial line distribution of Group 9 Element 3. Scale bars: **b** 500 μm, **b1**–**b3** 50 μm, **e**–**g** 20 μm, **g1** 10 μm

To illustrate the 3D tomographic capability of the wsFPDT method and to measure the lateral and axial resolution of the system quantitatively, we measured a phase resolution target (PRT) placed at a tilt, with its left side slightly padded up. The experimental setup is shown in Fig. 3a. Figure 3b demonstrates a full-FOV low-resolution intensity image captured at the wavelength of 450 nm. The set of typical resolution elements positioned closest to the sensor (the blue-boxed area in Fig. 3b) was selected as the reconstructed object. The insets of Fig. 3b display the diffraction patterns of the selected area recorded at incident wavelengths of *λ* = 450 nm, 800 nm, and 1150 nm, respectively. The 3D rendering image of the tilted PRT reconstructed with the wsFPDT method is displayed in Fig. 3c. From the *x* − *z* projection of the reconstruction result, it can be estimated that the tilt angle is approximately 8°, as illustrated in Fig. 3d. Because of the missing cone at low frequencies, the spectral support reveals the low axial resolution in the low-frequency region. The axial stretching of large bars and blocks in Groups 6-7 remains severe despite using the hybrid regularization, resulting in obvious axial artifacts in the projection of Fig. 3d. Three RI slices with different *z*-axis depths were extracted, clearly presenting the elements of Group 6 (*z* = 16.7 μm, Fig. 3e1), Groups 8-9 (*z* = −3.28 μm, Fig. 3e2), and Group 7 (*z* = −12.53 μm, Fig. 3e3), respectively. The complete layer-by-layer RI stacks and the corresponding 3D-rendered RI distribution of the phase resolution target are provided in Supplementary Video S1 for better visualization. As illustrated in Supplementary Video S1, the wsFPDT method effectively reveals the high-resolution 3D structure of the sample, demonstrating its 3D tomographic ability over a wide depth of field.

As discussed in Subsection “Non-interferometric ODT with wavelengthscanning”, the support for the accessible frequency coverage can theoretically evaluate lateral and axial resolution limits in the ODT framework. However, our method faces several challenges in practical implementations, including pixel aliasing and limited illumination angles. Therefore, we need to evaluate its imaging performance through experiments. We performed the wsFPDT method without the PSR algorithm (w/o PSR), which involves only the intensity constraint iteration using the intensity images without upsampling or pixel-binning, for RI reconstruction. Figure 3f shows the corresponding result, where Group 8 Element 3 (corresponding line width of 1.55 μm) is the smallest resolvable resolution unit (Fig. 3g). It indicates that without the PSR algorithm, the lateral resolution is limited by the sensor pixel size for lens-free imaging. As a comparison, Fig. 3h displays an enlarged view of the dashed-boxed area in Fig. 3e2, which is the result obtained using the PSR algorithm (w/ PSR). The line profile (Fig. 3i) shows the lateral resolution achieved by our wsFPDT as Group 9 Element 3 (corresponding to 775 nm line width), surpassing 2.15 times that of the theoretical Nyquist-Shannon sampling resolution limit imposed by the pixel size of the imaging sensor. Figure 3h1 presents the *y* − *z* slice across Group 9 Elements 1–3 corresponding to the dashed line in Fig. 3h. Figure 3j depicts the axial RI profile of Group 9 Element 3 with its full width at half maximum (FWHM) measured at 5.43 μm, indicating axial resolution. It is worth noting that the axial resolution depends on the lateral structure of the sample due to the optical transfer function (OTF) with inhomogeneous distribution in frequency space^14^. We also verified this phenomenon by simulations with microspheres of different diameters (Supplementary Note S3). Considering that common cells in biological laboratories, such as HeLa, C2C12, and diatoms, have diameters in the order of tens of micrometers, our system can provide 3D tomographic imaging for cell cultures with sufficient resolution. Especially for the subcellular features of interest, such as pseudopodia, wsFPDT can provide high-resolution reconstructions.

In general, lens-based ODT systems offer higher spatial resolutions with the angle-scanning scheme, mainly due to wider spectral coverage. However, this advantage does not apply to lens-free systems. As the sample’s diffraction patterns can be only captured on the defocus plane, oblique illumination produces two problems inevitably: (1) The diffraction patterns of the sample shift in response to variations in the illumination angle. Before the computation of tomographic reconstructions, it is imperative to align these patterns to a shared center using image registration algorithms, ensuring the accuracy of the resulting tomograms^52^. Also, the size of the diffraction pattern stretches along the tilt direction of illumination, and its deformation degree increases with the tilt angle, hindering sub-pixel accuracy level registration and reducing reconstruction resolution. (2) As shown in Fig. S5 in Supplementary Note S5, a large illumination angle can cause the image at the sensor edge to ‘fly out’ of the measurable FOV during acquisition, resulting in incomplete data recording. The reconstruction resolution is higher for samples located in the center and gradually decreases towards the edge of the FOV. However, the wsFPDT method produces diffraction images with minimal displacement. This allows for tomographic imaging with quasi-uniform resolution across the entire FOV. We conducted comparative experiments by placing the same test target in the center and edge regions of the sensor surface. The reconstructed results showed identical resolution, which is explained in detail in Supplementary Note S6. Furthermore, the experimental results of PRT demonstrate the ability of wsFPDT to achieve quasi-uniform and PSR RI recovery across the full FOV.

In this experiment, due to the PRT being tilted and suspended in the air, the selected target for reconstruction (indicated by the blue box in Fig. 3b) is located at a defocus distance of approximately 630 μm. When the target is placed directly on the sensor’s cover glass (Fig. S6 in Supplementary Note S6), the defocus distance is about 414 μm. The results of the two experiments show that the proposed wsFPDT method achieves a valid reconstruction depth-of-focus (DOF) of more than 200 μm without compromising the lateral imaging resolution (half-width of 775 nm). This is a significant improvement compared to the DOF of conventional lens-based ODT technologies, which typically range from five to ten times smaller^59,64^. The wsFPDT method holds great potential for tomographic imaging of biological samples with a certain volume, such as diatoms and common cells found in biological laboratories.

### Tomographic RI reconstruction of fixed diatom samples

**Fig. 4:**
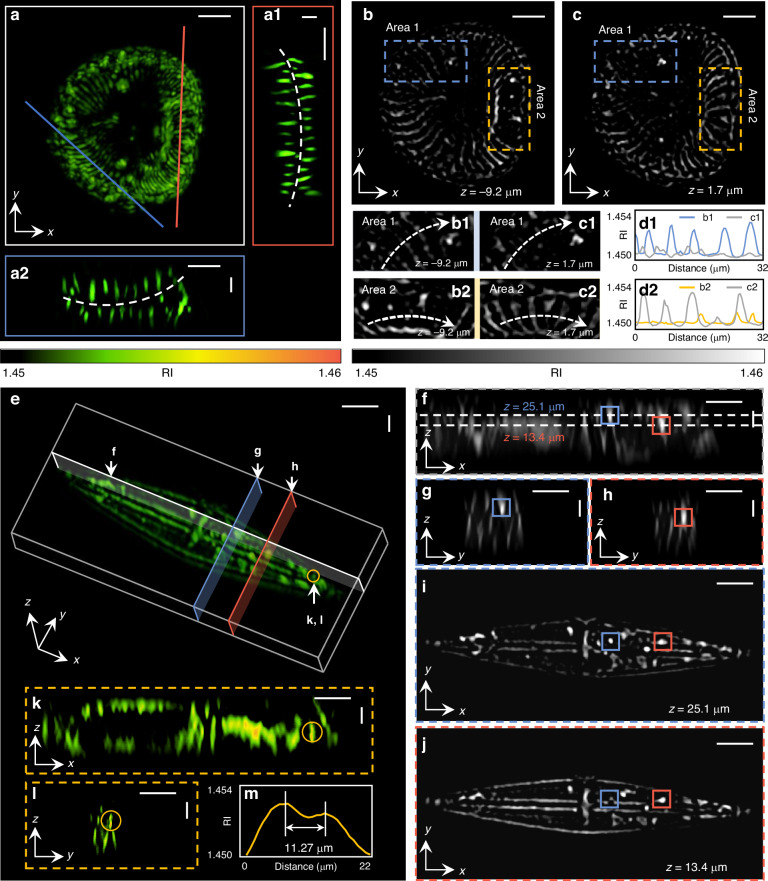
**3D RI reconstruction of diatoms using the proposed wsFPDT method**. **a** The *x* − *y* projection of rendered RI reconstruction for a *Campylodiscus hibernicus* diatom. **a1**, **a2** Two slices corresponding to the red and blue lines in **a**. **b**, **c** Two slices of 2D RI distribution images at *z* = −9.2 μm and *z* = 1.7 μm. **b1**, **b2** and (**c1**, **c2**) Zoom-in views of the two selected areas (Area 1 and Area 2) in **b** and (**c**). **d1**, **d2** RI line profiles corresponding to the lines in **b1** and **c1** (**b2** and **c2**). **e** 3D rendering results of the RI distribution of a *Naviculold* diatom. **f** The slice of *x* − *z* direction corresponding to the white line in **e**. **g**, **h** Two slices of *y* − *z* direction corresponding to the blue and red lines in **e**, respectively. **i**, **j** Two slices of RI distribution at different *z*-directional depths corresponding to the dashed lines in **f**. **k**, **l** The *x* − *z*, and *y* − *z* slices including the selected object marked by the yellow circle in **e**. **m** The RI line profile corresponding to the dashed line in **k**. Scale bar: 20 μm

To verify the effectiveness of wsFPDT in tomographic imaging of label-free and thick biological samples, we conducted 3D volumetric imaging of diatoms. In Fig. 4a, we present the reconstructed results of the RI distribution of a *Campylodiscus hibernicus* diatom projected in the *x* − *y* direction, revealing evenly distributed elongated stripes on the cell wall that converge towards the center of the diatom. Figure 4a1, a2 shows two vertical sections corresponding to the red and blue lines in Fig. 4a, exhibiting the overall saddle shape of the *C. hibernicus* diatom. Two RI slices of the *C. hibernicus* diatom at *z* = −9.2 μm and *z* = 1.7 μm are extracted and compared in Fig. 4b, c. Figure 4b1, c1 is the zoom-ins of the same sub-region (Area 1) in Fig. 4b, c, respectively. Similarly, Fig. 4b2, c2 enlarges the same sub-region (Area 2) shown in Fig. 4b, c, respectively. The RI values of certain coordinates (marked by dashed lines in Fig. 4b1, c1) within Area 1 at different axial positions are depicted in Fig. 4d1. Likewise, the RI values corresponding to the dashed lines in Fig. 4b2, c2 are illustrated in Fig. 4d2. Notably, the fine structure exhibits distinct characteristics within the same region at different axial positions. Besides, the recovered through-slice RI stacks of the *C. hibernicus* diatom and the 3D-rendered RI images are animated in Supplementary Video S2.

Figure 4e demonstrates the 3D rendering image of the RI of a *Naviculold* diatom. Furthermore, the recovered through-slice RI stacks of *Naviculold* diatom are animated in Supplementary Video S3. The *Naviculold* diatom has a narrow, boat-shaped appearance. For analysis, we selected two characteristic plastids which are marked with small blue and red boxes. As shown in Fig. 4f, the centers of these two selected plastids are located in the same *x* − *z* slice (marked with white lines in Fig. 4e). However, their centers are located at different axial depths, marked by the dashed lines (*z* = 25.1 μm and *z* = 13.4 μm), respectively. The RI slices at different *x*-axis positions, where the centers of these two selected plastids are located, are shown in Fig. 4g, h (marked with blue and red lines in Fig. 4e), respectively. Figure 4i, j depicts the *z*-axis slices at *z* = 25.1 μm and *z* = 13.4 μm, respectively, revealing diverse fine structures inside the diatom. Furthermore, two vertically adjacent plastids (marked by a yellow circle in Fig. 4e) were specifically selected to demonstrate the axial resolution of the wsFPDT method. Figure 4k, l shows the slices of *x* − *z* and *y* − *z* where the selected object center is located, respectively. In Fig. 4m, the RI line profile across the center of the fine structure indicates a spacing of 11.27 μm between these two plastids, further demonstrating wsFPDT can recover samples with an axial resolution of ~10 μm. In all these results, the appearance of distinct details at different sections can clearly be observed, demonstrating a powerful sectioning ability of wsFPDT. Thanks to large DOF, the wsFPDT method can perform high-resolution tomographic imaging on label-free, thick, and complex specimens, demonstrating its special advantages in imaging deep within complete samples, which has the potential to expand its application in various fields of biological research^65–67^.

### Tomographic RI reconstruction of fixed mouse mononuclear macrophage cells

**Fig. 5:**
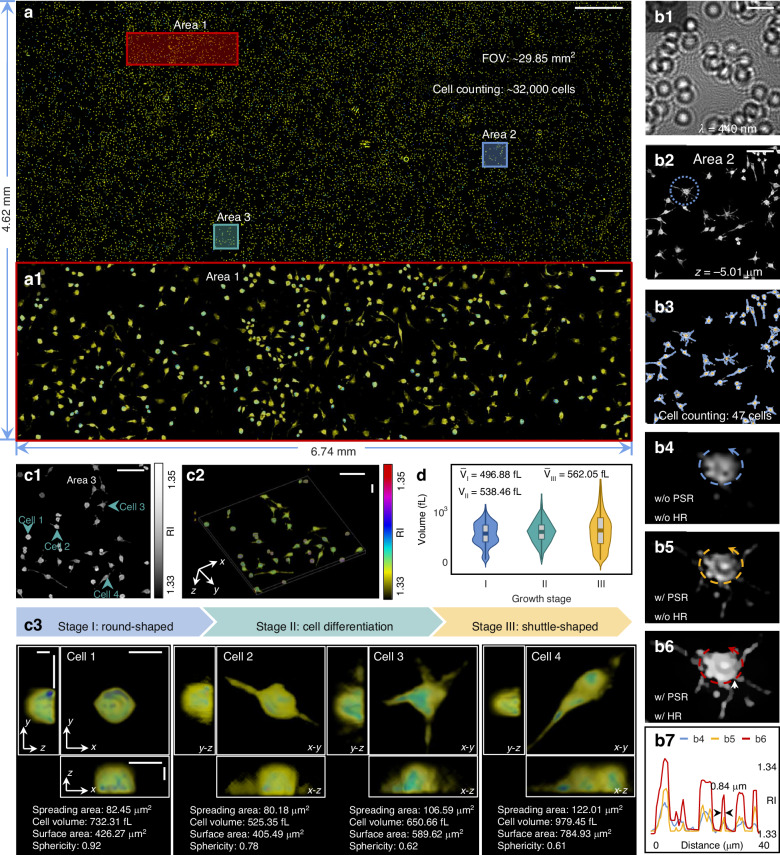
**Wide-FOV 3D imaging of a large population of mouse mononuclear macrophage cells based on the wsFPDT method**. **a** Full-FOV RI reconstruction of the fixed mouse mononuclear macrophage cells. **a1** The enlarged view of the red-boxed area (Area 1). **b1**, **b2** The captured image and the reconstructed RI slice at *z* = −5.01 μm corresponding to the blue-boxed area (Area 2) in **a**. **b3** Result after using the cell counting algorithm corresponding to **b2**. **b4**–**b6** Reconstruction results corresponding to the dashed area in **b2** under three different scenarios: without PSR (w/o PSR) and without hybrid regularization (w/o HR), with PSR (w/ PSR) but without hybrid regularization (w/o HR), with PSR (w/ PSR) and with hybrid regularization (w/ HR). **b7** RI line profiles labeled by the dashed lines in **b4**–**b6**. **c1** The reconstructed RI slice at *z* = −5.01 μm of the green-boxed sub-region (Area 3) in **a**. **c2** 3D rendering. **c3** Different cells belonging to the 3 typical growth stages and the corresponding morphological indices. **d** Cell volume distributions at 3 different growth stages. Scale bars: **a** 500 μm, **a1**, **b**, **c1**, **c2** 50 μm, **c3** 10 μm

To exemplify the ability of the wsFPDT to perform high-throughput 3D imaging of biological samples, we imaged a microscopy slide of mouse mononuclear macrophage cells. The wsFPDT can reconstruct the 3D structure of all cells over the full FOV, providing valuable insights in statistical significance into studying the structural characteristics of cells and the effects of internal and external stimuli on cellular functions, such as osmotic stress and drug treatment^68^. Figure 5a presents the RI reconstruction of mouse mononuclear macrophage cells for the entire FOV. To enhance visibility, the enlarged image of Area 1 is displayed in Fig. 5a1. Figure 5b1, b2 shows the enlarged diffraction patterns and the corresponding RI reconstruction slice for Area 2 in Fig. 5a. The dashed box in Fig. 5b2 indicates the region chosen for algorithm comparison. Specifically, Fig. 5b4–b6 illustrates the results obtained using different combinations of PSR and hybrid regularization methods: without PSR and without hybrid regularization (w/o PSR and w/o HR), with PSR but without hybrid regularization (w/ PSR and w/o HR), and with both PSR and hybrid regularization (w/ PSR and w/ HR). The line profile passing through all pseudopodia is displayed in Fig. 5b7. The PSR technique facilitates the retrieval of high-frequency information, while hybrid regularization is instrumental in edge preservation and contrast enhancement. Consequently, the wsFPDT iteration with PSR and hybrid regularization markedly enhances the visibility of morphological features (e.g., pseudopodia) and reveals some internal structural details of cells.

Figure 5c1 shows an enlarged view of the reconstructed RI slice corresponding to the green-boxed sub-region of the FOV (Area 3). The corresponding 3D rendering is depicted in Fig. 5c2. Mouse monocyte macrophage cells undergo morphological changes depending on their phagocytic activity and chemokine secretion. In their initial state (Stage I), they typically exhibit a round or oval shape. When they encounter and engulf antigens, they develop pseudopods due to chemokine-mediated differentiation (Stage II). Excessive phagocytosis of antigens further transforms the cell shape into a shuttle or long shuttle form, accompanied by an increase in cell size and elongated pseudopodia (Stage III). We selected four representative cells at different growth stages for magnification display in Fig. 5c3 and provided quantitative analysis of important morphological indices, including spreading area, cellular volume, surface area, and sphericity index. The spreading area represents the cell’s occupied area in a slice of the focusing plane, while the cell volume and surface area are determined by the number and distribution of voxels above a specific threshold after axial length correction. By using the 3D RI distribution of each cell, we conducted statistical analysis on the morphological parameters of all the cells across the full FOV. Figure 5d illustrates the classification of the cells into three distinct growth stages based on their sphericity indexes, and statistical analysis was conducted on their volume. The volume of the cells shows an increasing trend as they undergo differentiation. Fig. S4 in Supplementary Note S3 shows the axial slices of the cells, and the reconstruction results using wsFPDT show the details that suggest the internal RI distributions of the cells, which are consistent with the traditional ODT reconstruction results^69^. Supplementary Video S4 showcases the full-FOV, quasi-uniform, pixel-super-resolved, and high-throughput tomographic capability of the wsFPDT method through the full-FOV 3D recovered results of mouse mononuclear macrophage cells and the 3D renderings of the selected sub-regions. The full-FOV 3D reconstruction contains ~1.83 billion effective voxels of quantitative 3D RI data, covering about 32,000 cells over a 29.85 mm^2^ FOV. Biomedical research often relies on statistical analysis to draw confident conclusions^70,71^. The experiment substantiates that wsFPDT is a high-throughput, high-resolution 3D imaging technique, capable of acquiring abundant and trustworthy experimental data with minimal resource and temporal expenditure, thereby realizing the functional analysis of a large population of cells. By quantifying the morphological parameters of each cell across the full FOV, wsFPDT exhibits powerful tomography capabilities and has significant application prospects for high-throughput drug development and label-free pathological analysis^72–74^.

## Discussion

### Advantages of wsFPDT with non-interferometric measurements

It is worth mentioning that wavelength-scanning-based tomographic imaging methods have previously been demonstrated in lens-based imaging systems^57,75^. Traditional ODT methods rely on interferometric and holographic phase measurement techniques to obtain the complex amplitude of the scattered field. However, these interferometric-based measurement schemes result in complex systems that require careful calibration, are susceptible to coherent noise, and are affected by environmental instability. In the case of a compact LFOCM platform, achieving interferometric measurements is challenging due to space limitations for accommodating a separated reference beam. Nonetheless, this challenge is overcome by the wsFPDT method, which utilizes an iterative ptychographic reconstruction that does not rely on interference. This breakthrough allows for the first tomographic imaging on a lens-free system with wavelength-scanning illumination. With wsFPDT, phase retrieval, PSR reconstruction, and 3D synthetic aperture imaging can be simultaneously achieved. Moreover, non-interferometric measurement schemes preserve the advantages of LFOCM systems, including their simple design and compact size. Once industrialized, these systems can be easily incorporated into cell culture chambers, enabling continuous, dynamic, and high-resolution 3D tomographic imaging of cells. This technology offers a novel tool for biomedical research and clinical diagnosis.

### Considerations on dispersion effect

Dispersion is an intrinsic property of optical media. The RI of a medium is influenced by its absorption of radiation, as described by the Kramers–Kronig relationship^76^. In the case of normal dispersive media, especially transparent substances like water, oil, and cells, the absorption within the illumination range used in our experiment is minimal, resulting in a negligible change in RI. We can quantitatively estimate this decrease in RI within the wavelength range of 430–1200 nm based on Sellmeier’s formula (typically, *Δn*/*n*_*obj*_ ~1%)^77^. Consequently, the variation in the RI difference (*n*_*obj*_ − *n*_*m*_) between the sample and the surrounding medium can be almost negligible throughout the range of wavelength tuning^78,79^. Among the various ODT techniques, wavelength scanning has been empirically proven to be an effective and elegant method for achieving high-resolution tomographic imaging, offering advantages such as motion-free and rapid image acquisition. Numerous scientific studies have reported successful utilization of wavelength scanning for high-resolution tomography^53,57,79–83^. Therefore, the approaches employing wavelength-scanning illumination remain viable and applicable for RI measurement, even in the presence of dispersion.

### Further consideration about the resolution of the wsFPDT method

In the LFOCT experimental platform using the wsFPDT method, the sensor pixel size becomes a critical factor that limits the lateral resolution, analogous to the resolution analysis in traditional 2D lens-free QPI methods^23^. According to the Nyquist-Shannon sampling theorem, the resolution of holographic reconstruction is fundamentally limited by the sampling resolution of the imaging device, as the recorded holographic fringes remain unamplified. Consequently, the imaging sensors are unable to capture the holographic oscillations corresponding to the high spatial frequency details of the specimen due to spatial aliasing or undersampling. To overcome this challenge, we incorporated insights from the wavelength-scanning lens-free PSR method^32^ and the pixel-binning model of traditional FPM^17,18^ and FPDT^59^. Specifically, we accounted for the pixel-binning model during the intensity constraint step (Step 3 in Fig. 2) of the wsFPDT iteration, resulting in lateral pixel-super-resolved reconstruction results with greater than 2× enhancement (details in Fig. 3 in Subsection “Tomographic RI reconstruction of the tiltedphase resolution target” and Fig. S6 in Supplementary Note S6). Further, by measuring the point spread function (PSF) that characterizes the pixel response accurately, the LFOCT imaging system can be realistically modeled and its lateral resolution can be potentially enhanced^84^.

Thanks to the on-axis wavelength-scanning illumination, our LFOCT system offers motion-free and quasi-uniform 3D spatial resolution across the full FOV. However, the spectral coverage obtained is limited. Although combining tilted illuminations in the experimental design can optimize the axial resolution of the system, it also introduces several challenges, such as image distortion, image registration problems, and reduced resolution for samples located at the sensor’s edge (Supplementary Note S5). To address the issue of missing data at low frequencies in the 3D spectrum, regularization methods are employed to recover low-frequency components. Specifically, we utilize the hybrid regularization method, combining TV regularization with non-negativity constraint^14,85^, in this study. The simulated and experimental results confirm the effectiveness of the wsFPDT in 3D RI reconstruction. It should be noted that the OTF of wsFPDT is not uniformly distributed, resulting in a spatial frequency-dependent variation in resolution, especially for axial resolution^14,86^. Experimental measurements reveal that the highest axial resolution achievable is 5.43 μm, with a gradual decrease in axial resolution for larger sample sizes. Fortunately, the subcellular structures of interest of the biological cell samples are small for lens-free imaging. Thus wsFPDT holds the potential in label-free 3D biological imaging.

In the field of computational optical imaging, the imaging performance is significantly influenced by the reconstruction algorithm, particularly the regularization prior employed. TV regularization implicitly assumes that the sample is sparse or piecewise constant^87,88^, which is reasonable for most biological cell specimens in lens-free imaging scenarios. Nevertheless, artifacts will be introduced into the reconstruction results when the samples deviate from the assumptions or excessive TV regularization is applied^14^. Inspired by the recent advances in deep learning for solving the missing cone problems in ODT^89–91^, we will attempt to integrate deep learning-based methods with the wsFPDT framework to enhance the low-frequency signal recovery, mitigate the reconstruction artifacts, and improve the accuracy of RI measurements in future research.

### Comparison with the lens-based FPDT and SS-OCT method

The conventional FPDT approach^59,60^ is to mount an LED array on a commercial microscope platform and record the images of the sample at multiple illumination angles. In contrast, our wsFPDT method requires a simple experimental setup, using only an industrial camera combined with an on-axis wavelength-variable illumination source. Both methods, in principle, do not calculate the complex amplitude (both amplitude and phase) of the total field through interferometric or holographic techniques. Instead, they utilize the captured intensity images to update the scattering potential spectrum of the sample, subsequently obtaining the 3D RI distribution. In the conventional FPDT method, the overlap between the Ewald shells at different illumination angles forms an arc on the spherical shell. Sufficient angular illumination measurements are necessary to ensure substantial overlap of the Ewald shells in 3D Fourier space. Consequently, the elements in the LED array need to be arranged as closely as possible to guarantee overlap in the high- and low-frequency regions of the 3D object spectrum. In the wsFPDT method, the Ewald spherical shells corresponding to different wavelengths theoretically only intersect at the origin. However, due to the pixel-discrete nature of the 3D spherical shells in the reconstruction algorithm, there will be a certain area of overlap between pixelated spherical shells as long as the wavelength interval is sufficiently small (Fig. S3 in Supplementary Note S4 for more details). Hence, the reconstructed 3D spectrum is ensured to be continuous and complete with appropriate wavelength spacing.

Conceptually, the wavelength-scanning scheme used in the proposed wsFPDT method bears a close resemblance to the light source utilized in the swept-source optical coherence tomography (SS-OCT)^80,92,93^. Both methods reconstruct the 3D spectrum by analyzing signals acquired from the object at different wavelengths. SS-OCT achieves axial sectioning through coherence gating^94,95^, capturing back-scattered signals from various depths in biological tissues to generate a complete depth profile of sample reflectivity at the probe position (A-scan). To create a cross-sectional image (B-scan), the probe is laterally scanned across the sample. Comparing the frequency support of these two methods in Supplementary Table S2 reveals that the reflected field-based SS-OCT offers a broader spectral coverage and higher axial resolution. However, due to the absence of low-frequency components, SS-OCT can only recover dark-field signals but not resolve the RI distribution. Thereby, SS-OCT is applicable only to strongly scattering samples, not weakly scattering samples like biological cells. Moreover, the introduction of reference beams further complicates the system. Conversely, the wsFPDT method, based on transmission field measurements, is better suited for recovering the 3D RI distribution of unstained biological cells. The non-interferometric optical path structure and full-FOV one-time measurement characteristics facilitate motion-free 3D imaging with a simple setup. For a more detailed comparison between the wsFPDT and SS-OCT methods, please refer to Supplementary Note S7.

### Conclusions

In summary, we presented a novel wsFPDT method and demonstrated its application in high-throughput 3D imaging of biological samples with an associated LFOCT experimental platform. The wsFPDT method employs a single on-axis, wavelength-tunable source (range: 430–1200 nm) to illuminate the sample and records the under-sampled diffraction patterns at various wavelengths. An iterative ptychographic reconstruction was then applied to fill the 3D scattering potential spectrum, and the 3D RI distribution of the sample was recovered after a 3D inverse Fourier transform. The wavelength-scanning scheme not only avoids mechanical movements during image acquisition and precise alignment of the original images for data processing but also ensures quasi-uniform, pixel-super-resolved imaging across the full FOV of the sensor. Using wsFPDT, we achieved a lateral half-pitch resolution of 775 nm and an axial half-pitch resolution of 5.43 μm, with a maximum imaging depth exceeding 200 μm over a FOV of 29.85 mm^2^ based on a 1.67 μm pixel size sensor, creating an effective voxel size of 3.26 μm^3^ in a sample volume of 5.97 mm^3^. The effectiveness of the proposed wsFPDT method was demonstrated by imaging different types of biological samples, including diatoms and mouse mononuclear macrophage cells. The LFOCT experimental platform, utilizing wsFPDT, enables high-resolution imaging of thousands of cells across the full FOV, providing 3D morphological parameters of individual cells for analysis. With the ability of high-resolution, label-free imaging across a wide FOV, this high-throughput, high-content 3D microscopy demonstrates its remarkable potential and applicability in life sciences and biomedical research^96–98^.

## Materials and methods

### Sample preparation

The quantitative phase resolution target (Benchmark Technologies Corporation, U.S.) used for resolution quantification was fabricated on a glass substrate (Fig. 3, Supplementary Figs. S5 and S6). The diatom microalgae slide was prepared by Fisher Scientific Co. (Fig. 4). The mouse mononuclear macrophage cells (Fig. 5) fixed in glycerin gelatin were purchased from a biological laboratory at NJUST. The polystyrene microsphere samples were prepared in-house (Supplementary Fig. S2). The samples consisted of a mixture of micro polystyrene beads (Polysciences Inc., *n* = 1.594 at *λ* = 483 nm) of different diameters, mainly 3 μm and 5 μm. These beads were immersed in RI matching oil(E High Dispersion Series, *n* = 1.58, Cargille) to create the final sample slide.

### Imaging acquisition and analysis

In the experiment, the image acquisition process is controlled by custom-written C++ software. This software enables sequential recording of diffraction patterns at different wavelengths, ensuring precise synchronization between the sensor and the illumination source. The wavelength-scanning procedure is motion-free, allowing for the quick acquisition of 57 full-frame 16-bit raw images. It takes approximately 6 seconds to capture these images, considering the camera’s exposure time of 1/156 ms. This time includes both the wavelength tuning and the switching of RF channels for AOTF. The short image acquisition time is advantageous in capturing the migration and morphological changes of cells, which typically occur within minutes in cell culture. Further details are provided in Supplementary Note S4.

To achieve lateral super-resolution in data processing, we selected an upsampling factor of four. It should be noted that this method cannot offer a significant improvement in the reconstruction results with a larger upsampling factor. To recover the full-field results from the experimental data, we split the original image into 9 × 7 overlapping sub-regions (each sub-FOV of 500 × 500 pixels), with a minimum of 50 pixels of overlap on each side. After completing all sub-region calculations, the full-FOV high-resolution 3D tomography result (15488 × 11056 × 256) was created by using an alpha-blending stitching method. Our reconstructions were performed using MATLAB on a Windows-based workstation equipped with a 5.80-GHz central processing unit (Intel Core i9-13900K) and 128 GB of random-access memory. As for the total processing time, it takes about 30 minutes, which could be further reduced by implementing GPU acceleration rather than MATLAB. Then the 3D volume rendering was conducted with Fiji.

## Supplementary information

Supplemental information for Lens-free on-chip 3D microscopy based on wavelength-scanning Fourier ptychographic diffraction tomography

Video S1

Video S2

Video S3

Video S4

## Data Availability

All data are available in the main text or the supplementary materials from the corresponding author upon reasonable request.
