# Supplementary material for: Lens-free on-chip 3D microscopy based on wavelength-scanning Fourier ptychographic diffraction tomography
Source: Light Sci Appl. 2024 Sep 5;13:237. doi: 10.1038/s41377-024-01568-1 (PMC11377727; doi:10.1038/s41377-024-01568-1)
Supplement: Supplementary file 1 — Supplemental information for Lens-free on-chip 3D microscopy based on wavelength-scanning Fourier ptychographic diffraction tomography [file 41377_2024_1568_MOESM1_ESM.pdf]

Supplementary Information for
Lens-free on-chip 3D microscopy based on wavelength-scanning Fourier ptychographic diffraction tomography

Xuejuan Wu[1,2,3], Ning Zhou[1,2,3], Yang Chen[1,2,3], Jiasong Sun[1,2,3], Linpeng Lu[1,2,3], Qian Chen[3,], and Chao Zuo[1,2,3,*]**

[1]Smart Computational Imaging (SCI) Laboratory, Nanjing University of Science and Technology, Nanjing, Jiangsu Province 210094, China
[2]Smart Computational Imaging Research Institute (SCIRI) of Nanjing University of Science and Technology, Nanjing, Jiangsu Province 210094, China
[3]Jiangsu Key Laboratory of Spectral Imaging & Intelligent Sense, Nanjing University of Science and Technology, Nanjing, Jiangsu Province 210094, China
[*]zuochao@njust.edu.cn
[**]chenqian@njust.edu.cn

ABSTRACT

This document provides supplementary information for “lens-free on-chip 3D microscopy based on wavelength-scanning Fourier ptychography diffraction tomography”.

Contents

Supplementary Note S1. Forward model of wavelength-scanning Fourier ptychographic diffraction tomography (wsFPDT)
Supplementary Note S2. Detailed description of the reconstruction algorithm
Supplementary Note S3. Validation of the effectiveness of the wsFPDT method based on simulations and experiments
Supplementary Note S4. Analysis of spectral coverage in wsFPDT method
Supplementary Note S5. Analysis of multi-angle illumination experimental scheme in LFOCT systems
Supplementary Note S6. Illustration of wsFPDT method for quasi-uniform pixel-super-resolved tomography over the full FOV
Supplementary Note S7. Comparison of the wsFPDT method with the swept-source optical coherence tomography (SS-OCT) technique

Supplementary Note S1. Forward model of wavelength-scanning Fourier ptychographic diffraction tomography (wsFPDT)

When light propagates in non-uniform media, it is affected by differences in permittivities, leading to scattering of light in three-dimensional (3D) space[1]. The scattered light field emitted usually carries information on the refractive index (RI) distribution within the sample. In the basic theory of optical diffraction tomography (ODT)[2], scattering potential is commonly used to describe the 3D RI distribution of thick samples, and the expression is as follows:

$$V(\mathbf{r}) = k_0^2 \left[n^2(\mathbf{r}) - n_m^2 \right], \tag{S1}$$

where $k_0 = \frac{2\pi}{\lambda}$ is the wave number in the free space, $n(\mathbf{r})$ is the RI distribution of the sample space, and n_m is the RI of the surrounding medium (assumed to be constant). In this document, we use the vectors $\mathbf{r} \equiv (x, y, z) \equiv (\mathbf{r}_T, z)$ and $\mathbf{k} \equiv (k_x, k_y, k_z) \equiv (\mathbf{k}_T, k_z)$ to define the spatial coordinates and the corresponding frequency coordinates, respectively.

To simplify the model, we assume a monochromatic plane wave $U_{in}(\mathbf{r})$ irradiates a thick phase object (such as a biological cell). The resultant transmitted field $U(\mathbf{r})$ is the sum of the incident field, $U_{in}(\mathbf{r})$, and the scattered field, $U_s(\mathbf{r})$, i.e., $U(\mathbf{r}) = U_{in}(\mathbf{r}) + U_s(\mathbf{r})$. Among these, the propagation law of scattered light field can be described by the Helmholtz equation:

$$\left[\nabla^2 + k_m^2 \right] U_s(\mathbf{r}) = V(\mathbf{r}) U(\mathbf{r}), \tag{S2}$$

where $k_m = k_0 n_m$ is the wave number in the surrounding medium, $\nabla^2 = \frac{\partial^2}{\partial^2 x} + \frac{\partial^2}{\partial^2 y} + \frac{\partial^2}{\partial^2 z}$ is the 3D Laplacian operator. Equation (S2) establishes the relationship between the scattered field, $U_s(\mathbf{r})$, and the scattering potential, $V(\mathbf{r})$, of the sample. However, since $U_s(\mathbf{r})$ appears on both sides of Eq. (S2), it cannot be solved directly. To linearize the imaging model and obtain the analytical solution of Eq. (S2), two approximations, Born and Rytov, can usually be applied to find the first-order solution. These approximations aim to find the approximate solution by considering only a single scattering event.

When the RI values of the sample are close to its surrounding medium, the light scattering will be very weak. In this case, it can be assumed that the scattered field can be negligible compared to the incident field, i.e., $U_s(\mathbf{r}) \ll U_{in}(\mathbf{r})$, so that

$$U_{s1}(\mathbf{r}) \approx U_s(\mathbf{r}) = U(\mathbf{r}) - U_{in}(\mathbf{r}). \tag{S3}$$

Note that the first-order Born approximation[2] assumes that objects are weakly scattering, implying that sample-induced absorption is negligible and the total phase delay must be much smaller than $\pi/2$[3,4]. Indeed, the applicability of the Born approximation to the study of large phase objects is limited because it is largely restricted to the imaging of optically thin samples. When the sample is large or its RI much higher than that of the medium, the Rytov approximation is preferred to determine $U_{s1}(\mathbf{r})$.

The Rytov approximation[3,5] assumes that the total field has a complex phase function (e.g., $U(\mathbf{r}) = \exp[\phi(\mathbf{r})]$). The complex phase of the total field is the superposition of the complex phases of the incident

and scattered fields, expressed by the formula $\phi(\mathbf{r}) = \phi_{in}(\mathbf{r}) + \phi_s(\mathbf{r})$. When it is substituted into Eq. (S3), the first-order scattered field $U_{s1}(\mathbf{r})$ can be written as

$$U_{s1}(\mathbf{r}) \approx U_{in}(\mathbf{r})\{\exp[\phi_s(\mathbf{r})] - 1\} \approx U_{in}(\mathbf{r})\phi_s(\mathbf{r}) = U_{in}(\mathbf{r}) \ln\left[\frac{U(\mathbf{r})}{U_{in}(\mathbf{r})}\right]. \tag{S4}$$

In fact, if the scattered field is weak or the total sample-induced phase delay is small (i.e., $\phi_s(\mathbf{r}) = \phi(\mathbf{r}) - \phi_{in}(\mathbf{r}) \rightarrow 0$), the Rytov approximation can be reduced to Born approximation[4]:

$$\begin{aligned} U_{s1}^{R}(\mathbf{r}) &\approx U_{in}(\mathbf{r})\phi_s(\mathbf{r}) = U_{in}(\mathbf{r})[\phi(\mathbf{r}) - \phi_{in}(\mathbf{r})] \\ &\approx \exp[\phi_{in}(\mathbf{r})]\{\exp[\phi(\mathbf{r}) - \phi_{in}(\mathbf{r})] - 1\} \\ &= \exp[\phi(\mathbf{r})] - \exp[\phi_{in}(\mathbf{r})] \\ &= U(\mathbf{r}) - U_{in}(\mathbf{r}) = U_{s1}^{B}(\mathbf{r}), \end{aligned} \tag{S5}$$

where $U_{s1}^{R}(\mathbf{r})$ and $U_{s1}^{B}(\mathbf{r})$ represent the first-order scattering field under these two approximations, respectively. The Rytov approximation includes the Born approximation as a special case when the object is weakly scattering. The validity of the Rytov approximation does not depend on the total phase shift induced by the sample, but on the RI gradient within the sample. It has been demonstrated that the Rytov approximation is superior to the Born approximation for the imaging of thick biological samples[3].

Then, using the Green's function, the linear relationship between the first-order scattering field and the scattering potential can be derived:

$$U_{s1}(\mathbf{r}) = \int G(\mathbf{r} - \mathbf{r}')U_{in}(\mathbf{r}')d\mathbf{r}' = [V(\mathbf{r})U_{in}(\mathbf{r})] * G(\mathbf{r}), \tag{S6}$$

where $G(\mathbf{r})$ is the 3D Green's function in the surrounding medium ($G(\mathbf{r}) = \exp[jk_m|\mathbf{r}|]/4\pi|\mathbf{r}|$), $*$ is the 3D convolution. According to Eq. (S6), the first-order scattering field of the entire object is the superposition of the elemental fields of all point elements that constitute the object, which is equivalent to the convolution of the Green's function and the product of the objective function and the incident field. By applying a Fourier transform to both sides of Eq. (S6), we can derive a linear relationship between the first-order scattering field and the scattering potential of the object at the focusing plane ($z = 0$). This is the well-known Fourier diffraction theorem[2]:

$$\hat{V}(\mathbf{k} - \mathbf{k}_{in}) = -4\pi jk_z \hat{U}_{s1}(\mathbf{k}_T; z = 0)\delta(k_z - \sqrt{k_m^2 - |\mathbf{k}_T|^2}), \tag{S7}$$

where j is the imaginary unit, " $\hat{\cdot}$ " represents the Fourier spectrum of the corresponding variable. The constraint of $k_z - \sqrt{k_m^2 - |\mathbf{k}_T|^2}$ restricts the 3D frequency vector $\mathbf{k}$ to have values only on the Ewald shell, which means that the spectrum of the first-order scattering field $U_{s1}(\mathbf{r}_T; z = 0)$ corresponds to the Ewald shell with a radius of k_m. By changing the incident wavelength, the resulting first-order scattering field is mapped to different regions of the 3D spectrum of the sample, expanding the accessible frequency domain, which allows us to reconstruct the scattering potential of the sample.

Since only defocus intensity images can be captured in a lens-free on-chip tomography (LFOCT) system, z_D represents the defocus distance between the sample and the sensor. In Fourier space, the

propagation z_D distance of a light field along the optical axis can be simply expressed as the product of 2D first-order scattering potential and phase factor: $\hat{U}_{s1}(\mathbf{k}_T; z = z_D) = \exp(2\pi j k_z z_D)\hat{U}_{s1}(\mathbf{k}_T; z = 0)$. The Fourier diffraction theorem can be written as:

$$\hat{V}(\mathbf{k} - \mathbf{k}_{in}) = -j4\pi k_z \exp(-2\pi j k_z z_D)\hat{U}_{s1}(\mathbf{k}_T; z = z_D)\delta(k_z - \sqrt{k_m^2 - |\mathbf{k}_T|^2}) \tag{S8}$$

The solution of the inverse scattering problem requires the first-order scattering field $U_{s1}(\mathbf{r}_T)$ to be either measurable or obtainable by other means. Since intensity-only images are utilized to realize RI tomographic imaging, it is necessary to establish the relationship between measured intensity images and the scattering potential of samples. To simplify the description, the incident light field $U_{in}(\mathbf{r}_T)$ can be regarded as a plane wave with a unit amplitude. We use $U_{in}(\mathbf{r}_T)$ to normalize the scattered field and define three new variables $U_n(\mathbf{r}_T) = \frac{U(\mathbf{r}_T)}{U_{in}(\mathbf{r}_T)}$, $U_{sn}(\mathbf{r}_T) = \frac{U_s(\mathbf{r}_T)}{U_{in}(\mathbf{r}_T)}$, and $U_{s1n}(\mathbf{r}_T) = \frac{U_{s1}(\mathbf{r}_T)}{U_{in}(\mathbf{r}_T)}$. Then, combined with Eq. (S4), the normalized complex amplitude on the sensor plane can be written as:

$$U_n(\mathbf{r}_T; z = z_D) = \exp[U_{s1n}(\mathbf{r}_T; z = z_D)]. \tag{S9}$$

The relationship between the measurement field and the first-order scattering field is as follows:

$$I(\mathbf{r}_T; z = z_D) = |U_n(\mathbf{r}_T; z = z_D)|^2 = |\exp[U_{s1n}(\mathbf{r}_T; z = z_D)]|^2. \tag{S10}$$

During the experiment, the intensity image containing the sample information was divided by the background intensity to obtain a normalized value to ensure that the measurement data conformed to the formula. By combining Eqs. (S8) and (S10), a deterministic relationship can be established between the measured intensity $I(\mathbf{r}_T; z = z_D)$ and the scattering potential $V(\mathbf{r})$, resulting in the forward imaging model for wsFPDT.

Supplementary Note S2. Detailed description of the reconstruction algorithm

Based on the forward imaging model described in the first section of the supplementary materials, we apply the wsFPDT method to iteratively update the scattering potential spectrum of the sample with the measured intensity images as constraints, obtaining the 3D RI distribution of the sample. The detailed algorithm process is shown in Figure 2 of the main text. In the experiments, the illumination wavelength is adjusted according to the incremental sequence $\{\lambda^i, i = 1,2,...,N\}$ (N is the number of wavelengths), and the superscript i is used to denote that the image was captured at the i-th illumination wavelength. Accordingly, the image sensor records a series of low-resolution images $\{I^i_{cap}(\mathbf{r}_T; z = z_D)\}$. Then we divide the intensity image containing the sample information by the background intensity at the corresponding wavelength to obtain a holographic image sequence $\{I^i_{norm}(\mathbf{r}_T; z = z_D)\}$ with consistent intensity. This can compensate for the non-uniformity of the power spectrum distribution of the supercontinuum laser as well as the sensor response efficiency. Furthermore, the auto-focusing algorithm is used in advance to calculate the distance z_D between the sample and the sensor[6,7]. In addition, We assume that the RI of the background medium is a constant, denoted as n_m.

Step 1: Initialization. We initialize the sample into a uniformly distributed high-resolution RI space, $n(\mathbf{r})$. For the i-th wavelength λ^i, we calculate the scattering potential $V^i(\mathbf{r})$ using Eq. (S1) and perform a 3D Fourier transform on it to obtain its high-resolution initialization spectrum $\hat{V}^i(\mathbf{k})$. Thereafter, we sequentially take the images into the wsFPDT iterative reconstruction process, updating the initial guess of the scattering potential spectrum.

Step 2: Extraction of 3D sub-spectrum. For the selected illumination wavelength λ^i, the corresponding scattering potential spectrum is $\hat{V}^i(\mathbf{k})$. The 3D sub-spectrum at λ is extracted, which is the 2D Ewald spherical shell corresponding to $\mathbf{k} - \mathbf{k}^i_{in}$ in $\hat{V}^i(\mathbf{k})$ space. After projection along the k_z direction, we obtain the 2D sub-spectrum according to

$$\hat{U}^i_{s1n}(\mathbf{k}_T; z = z_D) = -\frac{j}{4\pi k_z} \exp(2\pi j k_z z_D) \hat{V}^i(\mathbf{k} - \mathbf{k}^i_{in}) \delta(k_z - \sqrt{{k^i_m}^2 - |\mathbf{k}_T|^2}). \tag{S11}$$

Inverse Fourier transform is taken on the resultant spectrum $\hat{U}^i_{s1n}(\mathbf{k}_T; z = z_D)$ to obtain normalized first-order scattered field $U^i_{s1n}(\mathbf{r}_T; z = z_D)$. Then, the normalized complex amplitude $U^i_n(\mathbf{r}_T; z = z_D)$ can be obtained using Eq. (S9).

Step 3: Intensity constraint. The low resolution intensity image $I^i_{norm}(\mathbf{r}_T; z = z_D)$ is used to update $I^i_n(\mathbf{r}_T; z = z_D) = \left|U^i_n(\mathbf{r}_T; z = z_D)\right|^2$ by introducing the pixel binning method[8]:

$$I^i_{change}(\mathbf{r}_T; z = z_D) = \frac{I^i_{norm\_M}(\mathbf{r}_T; z = z_D)}{I^i_{n\_bin}(\mathbf{r}_T; z = z_D)} I^i_n(\mathbf{r}_T; z = z_D), \tag{S12}$$

where $I^i_{norm\_M}(\mathbf{r}_T; z = z_D)$ is a version of $I^i_{norm}(\mathbf{r}_T; z = z_D)$ after interpolation, and $I^i_{n\_bin}(\mathbf{r}_T; z = z_D)$ is calculated by downsampling $I^i_n(\mathbf{r}_T; z = z_D)$ via $M \times M$ average pooling and then upsampling with the nearest neighborhood interpolation.

Next, we use a relaxation coefficient α to update the complex amplitude in the sensor plane[9]:

$$\bar{U}_n^i(\mathbf{r}_T; z = z_D) = \alpha\sqrt{I_{change}^i(\mathbf{r}_T; z = z_D)}\exp\{j\arg[U_n^i(\mathbf{r}_T; z = z_D)]\} + (1-\alpha)U_n^i(\mathbf{r}_T; z = z_D), \quad \text{(S13)}$$

where $\arg(\cdot)$ represents the function of obtaining the argument, α is the relaxation parameter controlling the amount of feedback from the previous estimate, and "$\bar{\cdot}$" represents the updated variable.

Finally, according to Eq. (S9), we convert the updated measured field $\bar{U}_n^i(\mathbf{r}_T; z = z_D)$ to the updated first-order scattering field:

$$\bar{U}_{s1n}^i(\mathbf{r}_T; z = z_D) = \ln[\bar{U}_n^i(\mathbf{r}_T; z = z_D)]. \quad \text{(S14)}$$

By performing the Fourier transform on the updated first-order scattering field $\bar{U}_{s1n}^i(\mathbf{r}_T; z = z_D)$, the updated two-dimensional sub-spectrum $\bar{\hat{U}}_{s1n}^i(\mathbf{k}_T; z = z_D)$ can be obtained.

Step 4: Update of 3D spectrum. Using Eq. (S8), $\bar{\hat{U}}_{s1n}^i(\mathbf{k}_T; z = z_D)$ is remapped to an Ewald spherical shell, and then inserted into its corresponding location of the original 3D spectrum $\hat{V}^i(\mathbf{k})$, thus completing the update of the scattering potential spectrum. It should be noted that, since the 3D scattering potential is wavelength-dependent, the 3D spectrum should be changed proportionally before moving to the next wavelength to update the image, i.e., $\hat{V}^{i+1}(\mathbf{k}) = (\lambda^i/\lambda^{i+1})^2\bar{\hat{V}}^i(\mathbf{k})$. The iterative process of **Steps 2 to 4** is repeated until the iteration of wsFPDT converges to all acquired intensity images (N images).

Step 5: Regularization. To minimize the artifact introduced by the missing angle space, we adopt a hybrid regularization method[10] that combines total variational (TV) regularization with non-negativity constraint to fill in the missing information in the scattering potential spectrum:

$$\begin{cases} V_{ret}^i(\mathbf{r}) \geq 0, \\ V_{ret}^i(\mathbf{r}) = \underset{V_{ret}^i}{\arg\min}\left\{\frac{1}{2}\left\|V_{ret}^i(\mathbf{r}) - V^i(\mathbf{r})\right\|^2 + \gamma\left\|V_{ret}^i(\mathbf{r})\right\|_{TV}\right\}, \\ \left\|V_{ret}^i(\mathbf{r})\right\|_{TV} = \sum_{xyz}\left\|\nabla V_{ret}^i(\mathbf{r})\right\|. \end{cases} \quad \text{(S15)}$$

After 3D inverse Fourier transform, the scattering potential of the object is reconstructed, and the corresponding 3D RI distribution can be used for unlabeled 3D imaging of the sample.

Supplementary Note S3. Validation of the effectiveness of the wsFPDT method based on simulations and experiments

We illustrate the effectiveness of reconstructing the RI of 3D samples using wsFPDT by simulations. To match the wavelength tuning range with the actual experimental system, we set the wavelength range of the illumination source to 430 - 1200 nm in the numerical simulation. As shown in Fig. S1a, a pure-phase microsphere with a diameter of 5 μm is placed at a distance of Z_2 = 400 μm from the sensor plane. The RI values of the microsphere and its surrounding medium are 1.594 and 1.58, respectively. Figure S1b displays the diffraction patterns of the microsphere at different illumination wavelengths. Figures S1c1 and c2 illustrate the central cross-sections of the RI spatial distribution in the $x-y$ and $y-z$ directions, respectively. The corresponding axial slice of the frequency support is shown in Fig. S1c3. Using wsFPDT, we reconstructed the RI distribution of the microsphere from the intensity images acquired at different wavelengths. Figures S1d1 and d2 show the transverse and axial slices of the recovered result, respectively. Figure S1d3 illustrates the axial slice of the corresponding frequency support. The comparison between Figs. S1c1 and d1 illustrate that the transverse contour of the microsphere maintains its original shape. However, when comparing Figs. S1c2 and d2, it can be found that there is an axial extension of the microsphere, which is caused by the large amount of missing data in the 3D spectrum. To address this, the hybrid regularization method was employed to fill the missing information in the spectrum computationally. As demonstrated in Figure S1e3, hybrid regularization can significantly compensate for low-frequency information, resulting in more accurate RI estimation during microsphere reconstruction and alleviating axial stretching.

Theoretically, the spatial resolution of ODT is defined as the maximum spatial frequency in the reconstructed Fourier spectrum[11]. Because the optical transfer function (OTF) of wsFPDT is not uniformly distributed, the resolution varies with spatial frequency[10]. Hence, we simulated a microsphere with a smaller diameter. The simulation results show that shrinking the microsphere size (Fig. S1g1, diameter of 2 μm) leads to an increase in the reconstructed RI and a reduction in axial stretching (Figs. S1g2-g3), suggesting that the axial resolution of the wsFPDT method improves at relatively high frequencies. Figure S1g4 shows the reconstructed RI curve as a function of microsphere size, indicating that the wsFPDT method has higher accuracy in measuring the RI of high-frequency samples. When the diameter of the simulated microspheres is reduced to 0.5 μm, the RI reconstruction value is 1.5937, which shows a high degree of agreement with the true value (1.594) set in the simulation, with an error of 0.0003. Furthermore, to assess the anti-noise performance of the algorithm, we further conducted simulations under different noise conditions. Figure S1h1 shows the raw intensity images and the corresponding reconstruction results with 5%, 15%, and 25% Gaussian noise, respectively. Analyzing the sample RI on the central $x-y$ cross-section of the reconstructed results, we found that the reconstruction error remains small even at higher noise levels. For example, under 30% noise, the maximum RI reconstruction error does not exceed $\pm$ 0.0002. Simulations were repeated thirty times independently for each noise level. Overall, as the noise increases, the error of the reconstructed RI tends to increase, with only a mild reduction in

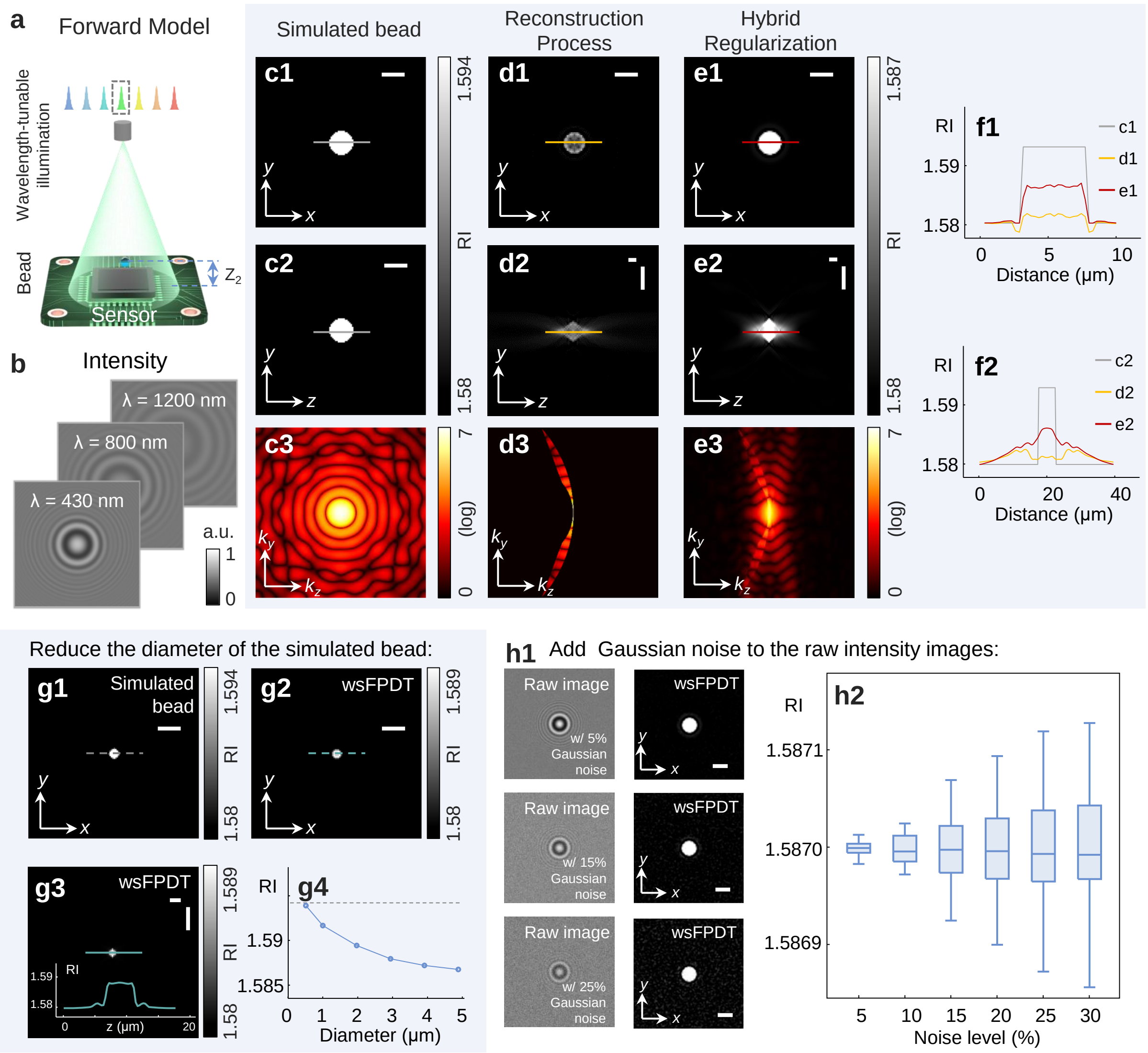

Figure S1. **a** A schematic diagram of the simulation model. **b** Diffraction patterns of the forward model simulation. **c1**, **c2** Central slices of the model RI. **c3** Axial slice of the 3D scattering potential spectrum. **d1-d3** Simulation results using the wsFPDT method without (w/o) the hybrid regularization. **e1-e3** Simulation results with (w/) the hybrid regularization. **f1** The distribution of RI values along the lines in **c1**, **d1**, and **e1**. **f2** The distribution of RI values along the lines in **c2**, **d2**, and **e2**. **g1-g3** The simulation results of a microsphere with a diameter of 2 μm. **g4** Variation of reconstructed RI with diameter. **h1-h2** Simulation results with different noise levels. Scale bar: 5 μm.

the mean (Fig. S1h2). Consequently, the wsFPDT method demonstrates the ability to reconstruct 3D RI distributions quantitatively with high fidelity.

To evaluate the 3D reconstruction capability of wsFPDT for complex samples, we simulated a synthetic cell phantom with inhomogeneous RI and fine structures as the test sample. Figure S2a shows the 3D view of the cell phantom. Figure S2b illustrates a $y-z$ cross-section containing two lipids (diameter 2.5 μm) distributed along the z-axis. As mentioned above, the axial resolution of the system varies with spatial frequency due to the inhomogeneous distribution of the OTF in frequency space[10]. Here to match the reconstructed axial stretch of the lipids (∼5.5 μm), we set the interval to 11 μm to ensure that they can be resolved exactly in the reconstruction. The $x-y$ slice in Fig. S2c reveals horizontally distributed bars. The 3D view of the reconstruction result is demonstrated in Fig. S2d. The results of the RI reconstructed without hybrid regularization show a severe elongation of the axial features due to spectral missing (Fig. S2e), while the lateral resolution is significantly better than the axial resolution (Fig. S2f). Figures S2g and h show that the reconstruction results of axial features are greatly improved and artifacts are reduced with hybrid regularization. It is apparent from the RI curves in Figs. S2i and j that the wsFPDT method allows the reconstruction of the fine structures located in both axial and transverse directions. Thus, wsFPDT is capable of tomographic imaging samples with complex 3D structures.

To verify the effectiveness of the wsFPDT method in practical applications, we used micro-sized microspheres as samples for our experiments. Specifically, the mixture of polystyrene microspheres (Polysciences Inc., n = 1.594 at λ = 483 nm) of different diameters (mainly 3 μm and 5 μm) are immersed in RI matching oil (E High Dispersion Series, n = 1.58, Cargille). Figure S3a demonstrates the raw image acquired directly by the image sensor. To demonstrate the quasi-uniform, high-throughput 3D tomographic capability of wsFPDT, we reconstructed three regions from the full field-of-view (FOV): the upper left corner, the central region, and the right edge, marked with blue, red, and green boxes, respectively. Figures S3b-d display the enlarged diffraction patterns of the three selected regions, respectively. Figures S3b1-d1 show the reconstructed RI slices at the focus plane, respectively. We selected microspheres with diameters around 5 μm in each of these three results for analysis, marked by blue, red, and green circles. The $x-y$ and $x-z$ slices containing the centers of the selected microspheres are shown enlarged in Figs. S3b2-d2 and b3-d3 for analysis. The RI values along the x and z directions are depicted in Fig. S3e. It can be seen that the wsFPDT method recovers the shape of the microspheres well in the transverse direction, and there is a large extension in the axial direction, which is basically consistent with the above theoretical analysis. However, the reconstruction results show that the axial stretch of the beads is somewhat worse than the simulation results, which may be the result of a convolution between the PSF and the bead shape. Compared to the reconstruction results in the central region of the sensor (marked with the red box), there is no significant degradation in the imaging results in the edge region (marked with the blue and green boxes). These experimental results indicate the wsFPDT method provides quasi-uniform lateral and axial resolution for 3D tomography across the full FOV.

Furthermore, we also selected two smaller microspheres and analyzed the reconstruction results (Figs. S3f-g). Figures S3f1-f2 (Figures S3g1-g2) show the RI values along the lateral and axial directions corresponding to the dashed lines in Fig. S3f (Fig. S3g). After 3D reconstruction on microspheres over the full

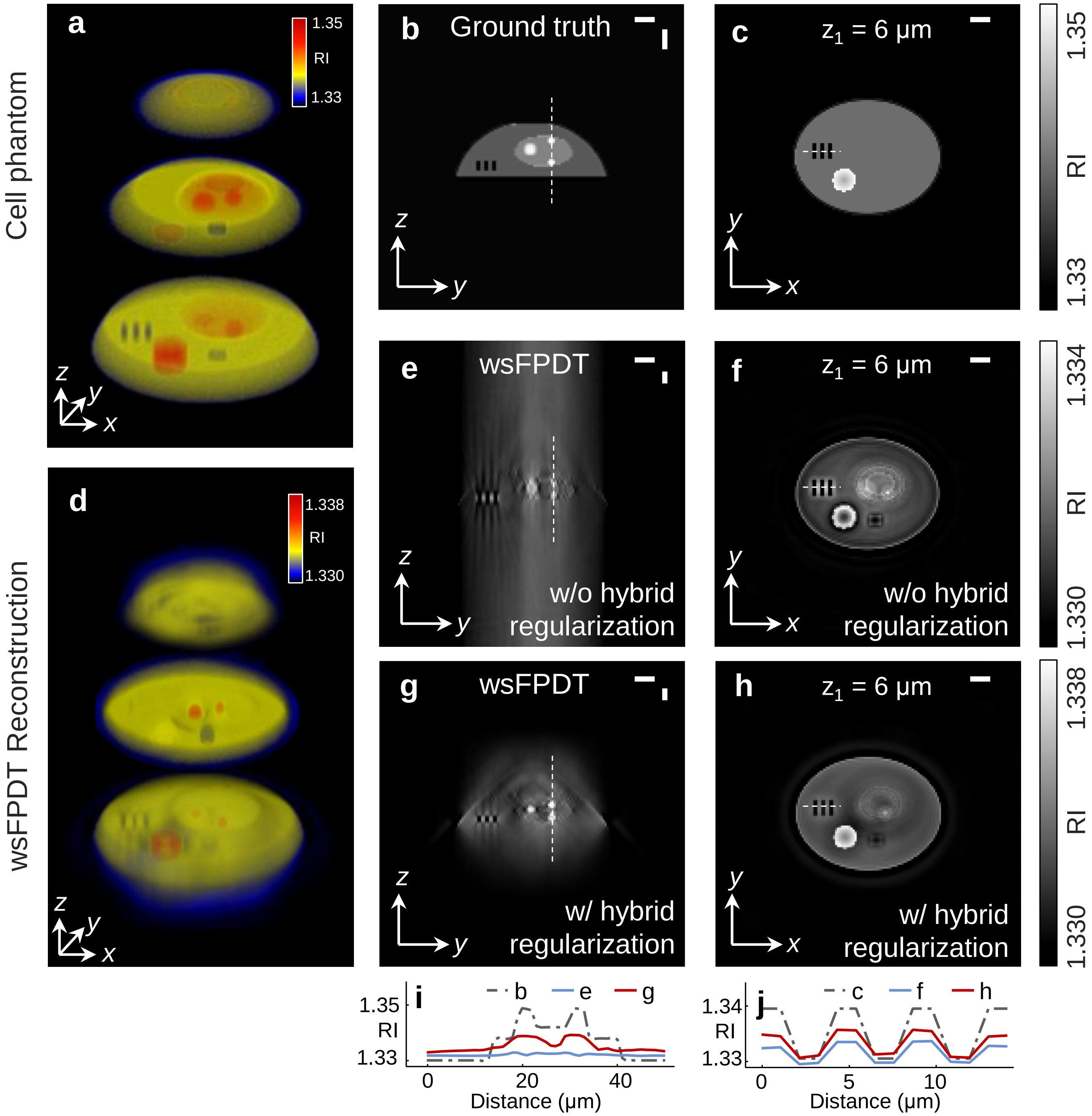

Figure S2. Reconstruction results of a cell phantom using wsFPDT. a 3D view of the cell phantom. **b**, **c** $y-z$ slice and $x-y$ slices extracted from **a**. **d** 3D view of the reconstruction result. **e**, **f** The $y-z$ and $x-y$ slices of the RI reconstructed by wsFPDT without hybrid regularization. Similarly, **g**, **h** The $y-z$ and $x-y$ slices of the RI reconstructed with hybrid regularization. **i** The distribution of RI values along the dashed lines in **b**, **e**, and **g**. **j** The distribution of RI values along the dashed lines in **c**, **f**, and **h**. Scale bar: 10 μm.

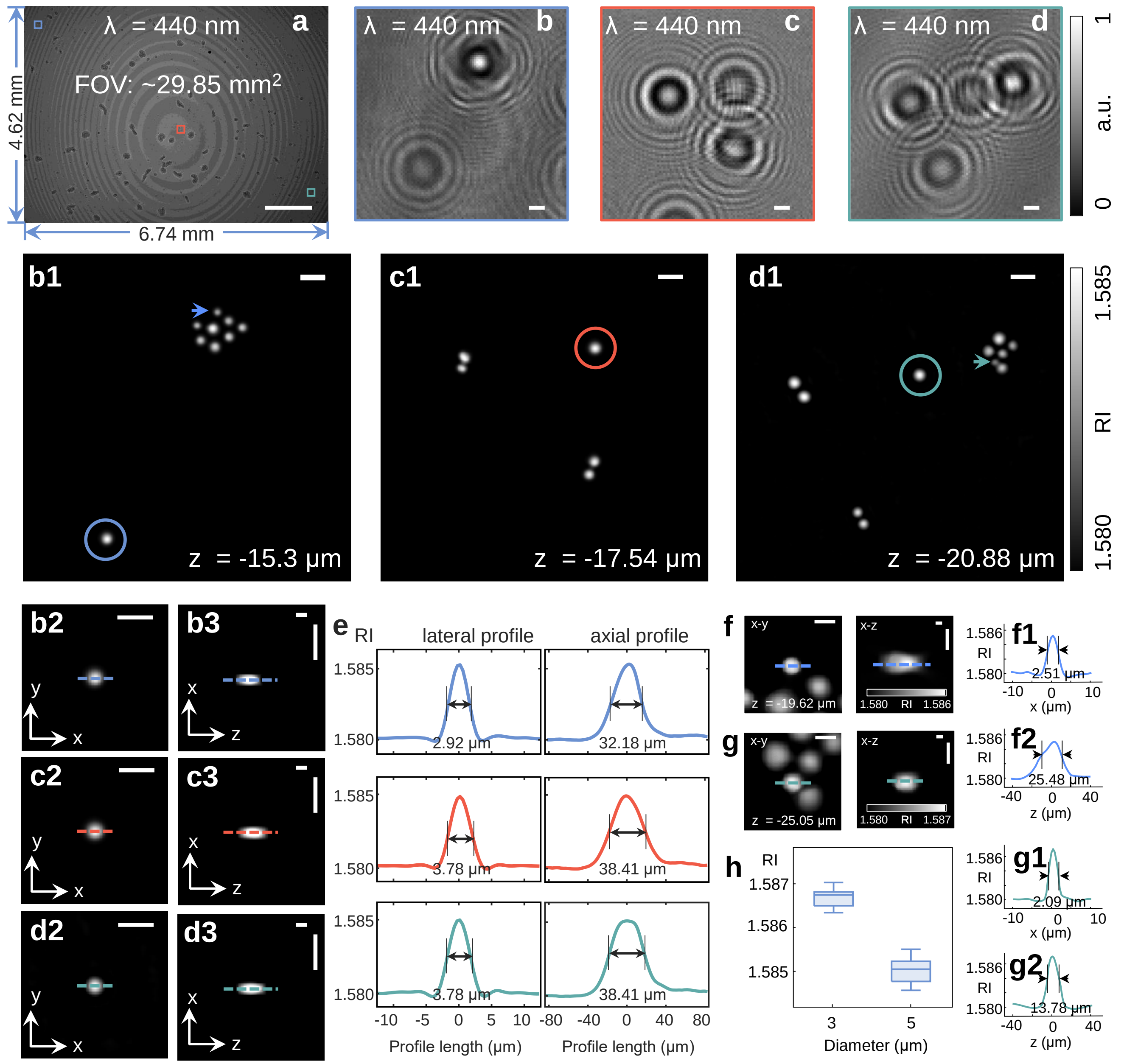

Figure S3. Experimental results of micro-polystyrene beads. a Raw intensity image acquired by the image sensor at the illumination wavelength λ = 440 nm. **b-d** Enlarged displays corresponding to the three boxed regions in blue, red, and green in **a**. **b1-d1** Reconstruction results of the three regions in **b-d**. **b2-b3** $x-y$ and $x-z$ slices where the center of the microsphere selected by the circle in **b1** is located. Similarly, **c2-c3** (**d2-d3**) The $x-y$ and $x-z$ slices where the centers of the selected microspheres in **c1** (**d1**), respectively. **e** The distribution of RI values along the lateral and axial directions, corresponding to the blue, red, and green lines in **b2**, **c2**, **d2**, **b3**, **c3**, and **d3**, respectively. **f-g** Enlarged displays and RI line profiles of two smaller microspheres marked with arrows in **b1** and **d1**, respectively. **h** The reconstructed RI results for 3 μm and 5 μm microspheres. Scale bars: **a** 1 mm, **b-d** 10 μm, **f-g** 5 μm.

FOV, we selected thirty 3 μm microspheres and thirty 5 μm microspheres each and statistically analyzed the reconstructed RI (Fig. S3h). The statistical results show that the mean value of the reconstructed RI for 3 μm microspheres is 1.5865, which is 0.0015 higher than the reconstruction result of 1.585 for 5 μm microspheres. As the sample size decreases, the axial stretching of the reconstruction is alleviated and the accuracy of the RI is improved.

Figure S4 demonstrates the 3D RI reconstruction of mouse mononuclear macrophage cells using the wsFPDT method. Figure S4a shows the 3D rendering of a round cell, with different directional slices shown in Figs. S4a1-a3. Figures S4b and c reveal similar results for the other two cells of different morphologies. RI slices suggest that RI is not uniformly distributed within the cell. Areas with a high RI indicate biological structures, while areas with a low RI may be areas of cytoplasmic distribution. The consistency between the results reconstructed by wsFPDT and the RI distribution measured by the ODT method using a 60× objective demonstrates the feasibility of the proposed method[12].

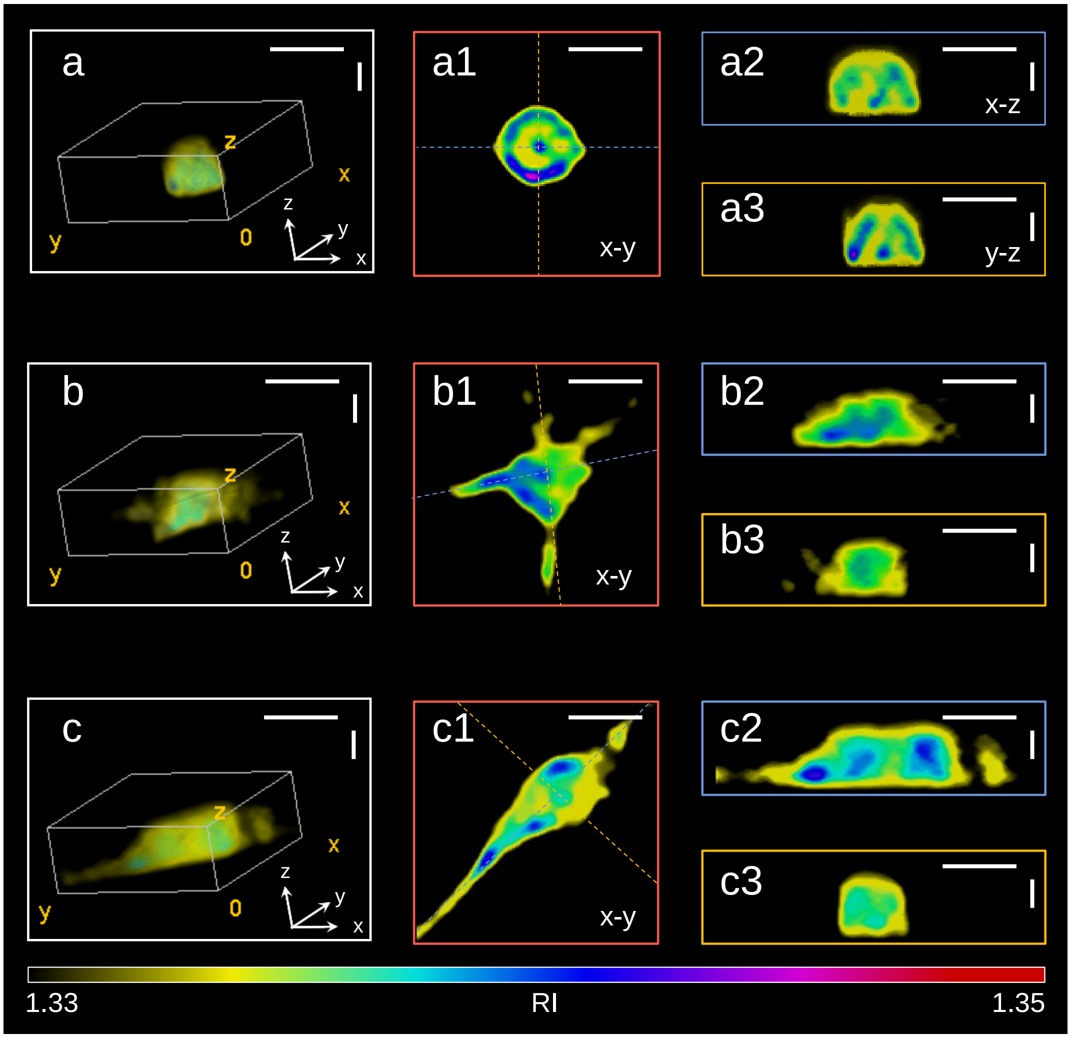

Figure S4. **Experimental results of mouse mononuclear macrophage cells. a-c** 3D rendering of 3 different cells. **a1-c1** The $x-y$ slices of RI distribution in **a-c**. **a2-c2** The slices corresponding to the blue lines in **a1-c1**. **a3-c3** The slices corresponding to the yellow lines in **a1-c1**. Scale bars: 10 μm.

Supplementary Note S4. Analysis of spectral coverage in wsFPDT method

S4.1 The number of input images

In wsFPDT, the vertices of Ewald spectra at different wavelengths are located at the origin of the frequency domain. During iterative reconstruction, it can be used for updating due to the enough overlap of frequency domain spherical caps corresponding to adjacent wavelengths after digitization (Fig. S5a). To achieve efficient and effective sample reconstruction, we need to determine the minimum number of incident wavelengths that guarantee spectral filling, minimize the number of images captured in the experiment, and enhance computational efficiency. We explored the effect of the number of raw images collected on the experimental results in a fixed wavelength range (430 - 1200 nm). We still use a pure phase microsphere with a diameter of 5 μm as a simulation model. The spectral support domains and the corresponding RI reconstruction results for the number of input images of 6, 21 and 57 are presented in Figs. S5b, c and d, respectively. Comparing Figs. S5b1, c1 and d1, it can be found that the spectral coverage increases as the number of images increases. Correspondingly, the axial stretching of the microsphere is alleviated (Figs. S5b3, c3 and d3) and the reconstructed RI values are increased. Figures S5e and f show the variation of axial stretch length and RI maximum with the change of the number of images, respectively. The axial stretch length of the microsphere converges when the number of images is greater than 42, and the RI size that can be recovered also converges when the number of images is not less than 57.

In the experiments, to ensure the continuity of the spectrum while reducing the computational effort, we acquired 57 raw images, which took about 6 seconds (including the exposure time of the camera, the time to tune the wavelengths and switch the RF channels of AOTF). To obtain full-field recovery results, when processing the experimental data, we usually split the original image into 9×7 sub-regions (each sub-FOV of 500×500 pixels), with at least 50 pixels of overlap on each side of the adjacent sub-regions. By using parallel computing and GPU acceleration, a full-FOV high-resolution 3D tomography result ($15488 \times 11056 \times 256$) is created by using an alpha-blending stitching method with the total computation time about 30 minutes.

S4.2 The influence of wavelength-scanning range on axial resolution

It is known that the radius of the Ewald spherical shell decreases as the wavelength increases so that the corresponding complex amplitudes at long wavelengths fill the low-frequency region of the spectrum, as is shown in Fig. S6a. Consistent with the previous simulations, a microsphere with a diameter of 5 μm is still used as the simulation object. The first row of Fig. S6b shows the results of the spectrum support domain and the corresponding RI reconstruction for the wavelength range of 430 - 1200 nm. The frequency support of the wsFPDT method is very narrow at low-frequency domain, resulting in a limited recovery of the low-frequency signal of the sample, which makes the RI reconstruction results of the microsphere samples appear “double-peak” in the axial direction. With only the non-negativity constraint, the “double-peak” phenomenon still exists (the 2nd row). After the hybrid regularization method combining TV regularization with non-negativity constraint (the 3rd row), the low-frequency

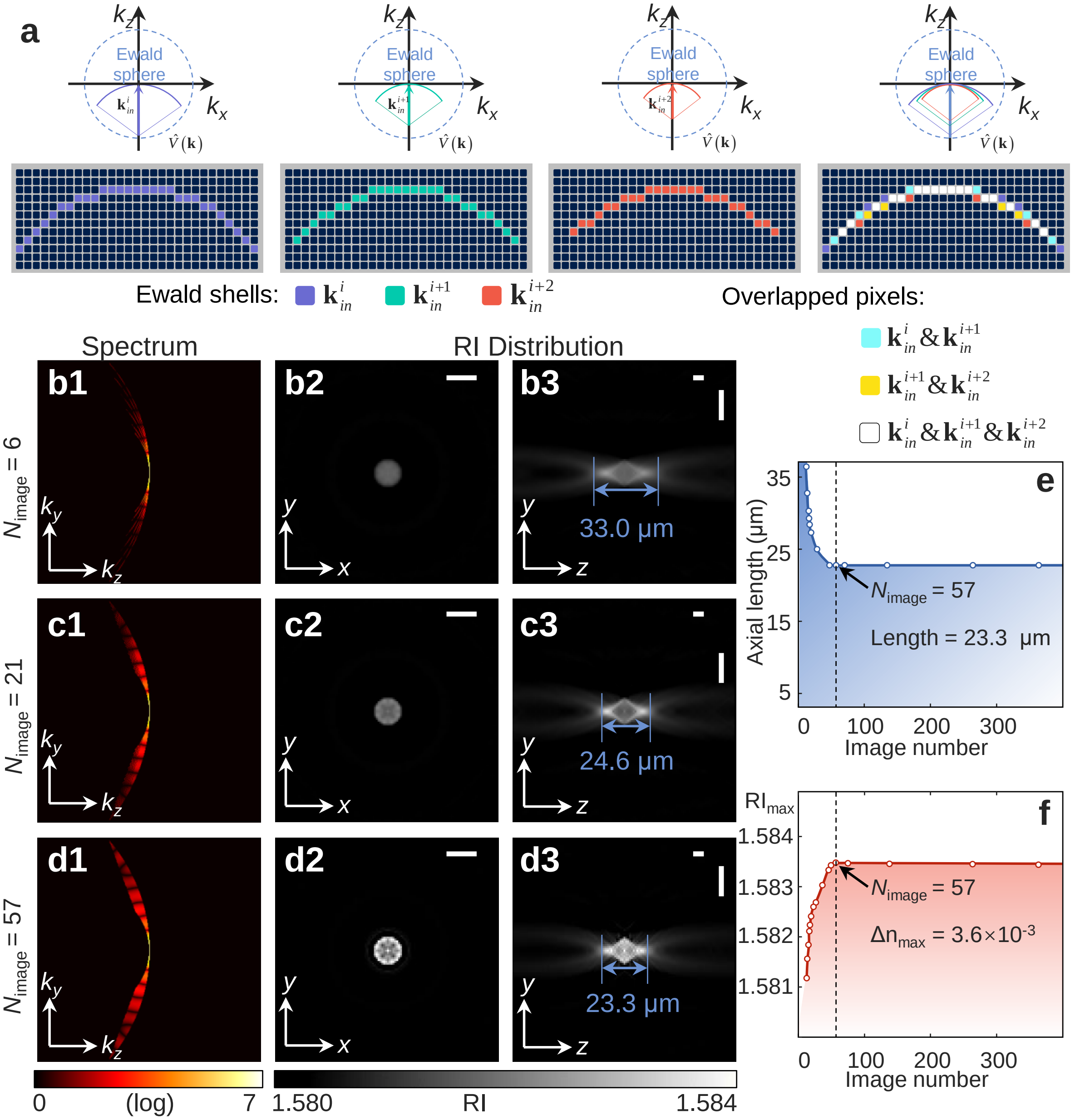

Figure S5. The frequency support and RI reconstruction results for a different number of input images in the wavelength range of 430 - 1200 nm. a The sub-spectrum coverage of wsFPDT. **b**, **c** and **d** The frequency support and the corresponding RI central slices for the input image numbers of 6, 21 and 57, respectively. **b1**, **c1** and **d1** The central slices of the frequency support. **b2**, **c2** and **d2** Transverse center slices of the RI distribution. **b3**, **c3**, and **d3** Axial center slices of the RI distribution. **e**, **f** The axial stretch length and the variation of the RI maximum with the number of input images, respectively. Scale bar: 5 μm.

part of the spectrum is well-filled, resulting in an increase in RI value and the disappearance of the axial "double-peak" phenomenon. Then, we explored the effect of the upper limit of the wavelength range on the axial resolution limit through simulation. It can be found that as soon as the upper limit of the wavelength scan range reaches 3.5 μm, the "double-peak" phenomenon basically disappears (the 1st row Fig. S6c) without any regularization methods. Simultaneously, the reconstruction results show a smaller axial stretch length of the bead and an increase in the RI value. Additionally, the axial stretch will shrink further after the hybrid regularization method. The low-frequency part of the 3D spectrum can be somewhat filled by raising the upper limit of the wavelength range. Nevertheless, the axial elongation and small RI valuation caused by the missing cone problem cannot be avoided.

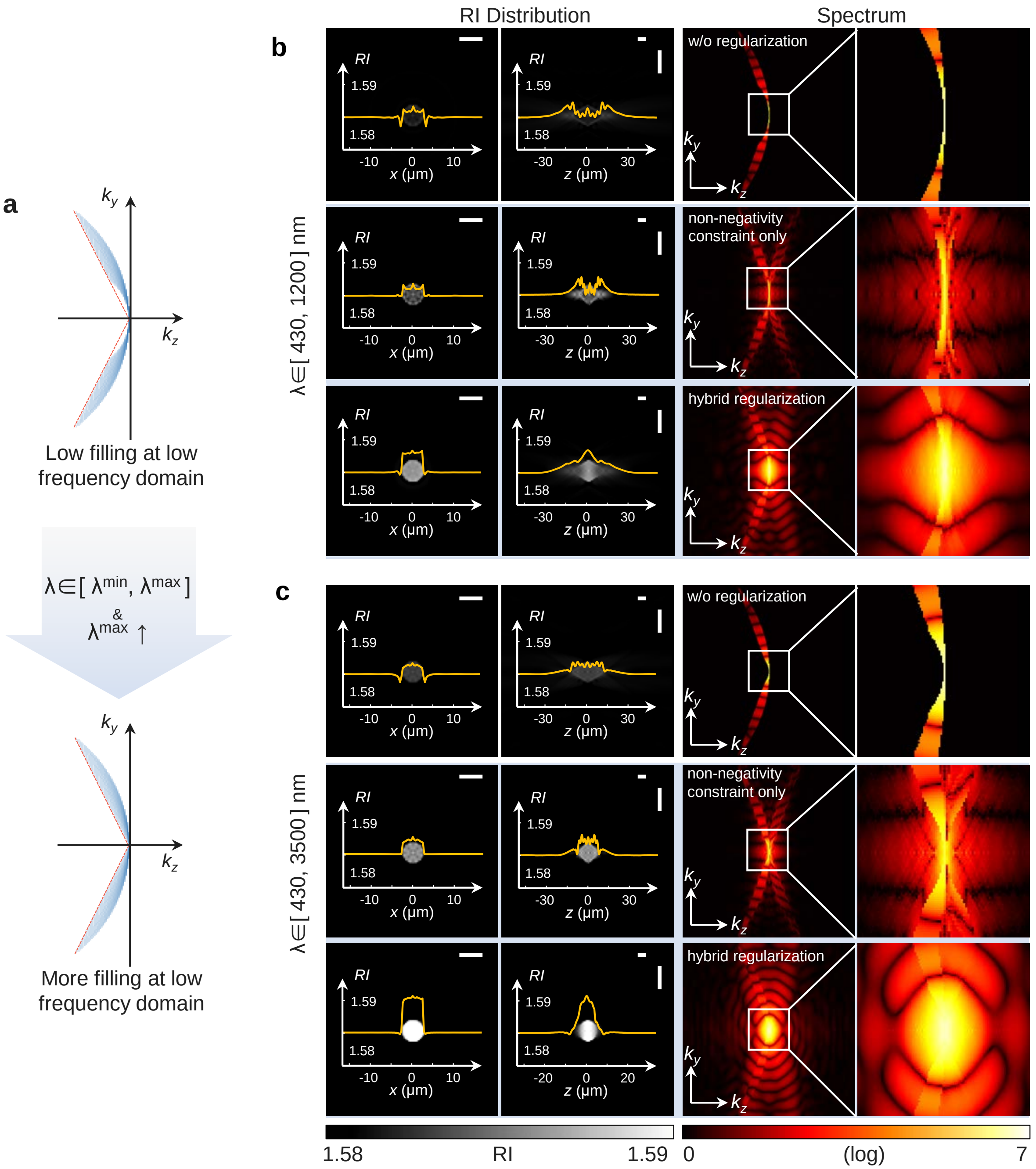

Figure S6. **Effect of the upper limit of wavelength range on the reconstruction results. a** (Schematic diagram) Increasing the upper limit of wavelength range on the filling of the low-frequency part of the spectrum. **b-c** RI reconstruction results and frequency supports corresponding to the wavelength ranges of 430 - 1200 nm and 430 - 3500 nm, respectively. The 1st row is reconstructed spectrum and RI slices without any constraint, the 2nd and 3rd row are the results after only non-negativity constraint and the hybrid regularization method for comparison. Scale bar: 5 μm.

Supplementary Note S5. Analysis of multi-angle illumination experimental scheme in LFOCT systems

The optical diffraction tomography (ODT) experiments[13,14] based on the use of conventional microscope platforms typically use multi-angle illumination schemes to obtain a doughnut-shaped transfer function, resulting in higher axial (and lateral) resolution. However, it is difficult to use an experimental scheme with multi-angle illumination in a lens-free system to obtain high-resolution 3D imaging over a large FOV. In the following, we will illustrate this with specific experiments.

First, the distance between the sensor and the sample is usually a few hundred microns or even 1 mm in LFOCT systems[15,16]. As shown in Fig. S7a, according to instructions in geometrical optics, which is fully based on Fermat's principle, the imaging position of the sample changes with the illumination angle. We designed a simple multi-angle illumination structure (as shown in Fig. S7b) to illustrate a common phenomenon of missing data caused by images 'flying out' of the measurable FOV of the sensor under a large-angle illumination. As shown in Fig. S7c, this simple angle-variable illumination system consists of a 3D-printed multi-angle mount (containing 20 fixed angles with a 10° angle interval) and a single-mode fiber-coupled illumination source (LP660-SF20, Thorlabs, the United States). In the experiment with only the illumination angle changing, Figures S7d-g show the diffraction images of the fixed sample captured at 4 different illumination angles (5°, 25°, 45° and 65°), respectively. To observe the imaging behavior at the edge of the sensor, we select the area marked by the blue box. As the illumination angle increases, the image 'flies out' of the sensor's measurable range (as shown in Figs. S7d1-g1). According to the geometric relations, if an LFOCT system (defocus distance $Z_2 \sim 400$ μm) has a large angle illumination of more than 60°, the image of the object will be off by more than 600 μm. Due to the loss of information, in a lens-free 3D imaging system based on multi-angle illumination, the imaging resolution decreases in the annular region near the edge of the sensor. The movement of the sample imaging position must be pulled back to the correct position by projection and alignment algorithms, and this is a complex and time-consuming operation.

Moreover, we choose a Siemens star as the observation target (marked with the red dashed box) and can find that it gradually elongates along the illumination direction as the illumination angle increases (as shown in Figs. S7d2-g2). This indicates that the angularly varying illumination also causes distortions in the imaging process, which creates problems in image alignment and requires additional calibration procedures[17]. For simple sparsely distributed objects, region selection can be performed based on the center of the object imaged[18]. However, as shown in Figs. S7h and i, for complex objects (e.g., USAF target), the increased illumination angle results in diffraction patterns of USAF targets that are not only significantly distorted but may be mixed with diffraction rings of other targets. So the image distortion imaging center is difficult to be determined. And the down-sampling process of the diffraction patterns by sensor imaging exacerbates the signal aliasing. The aforementioned reasons make the selection of the reconstruction area difficult and aggravate the difficulty of image registration. The use of only on-axis illumination provides the prerequisite for the wsFPDT method to perfectly circumvent these problems

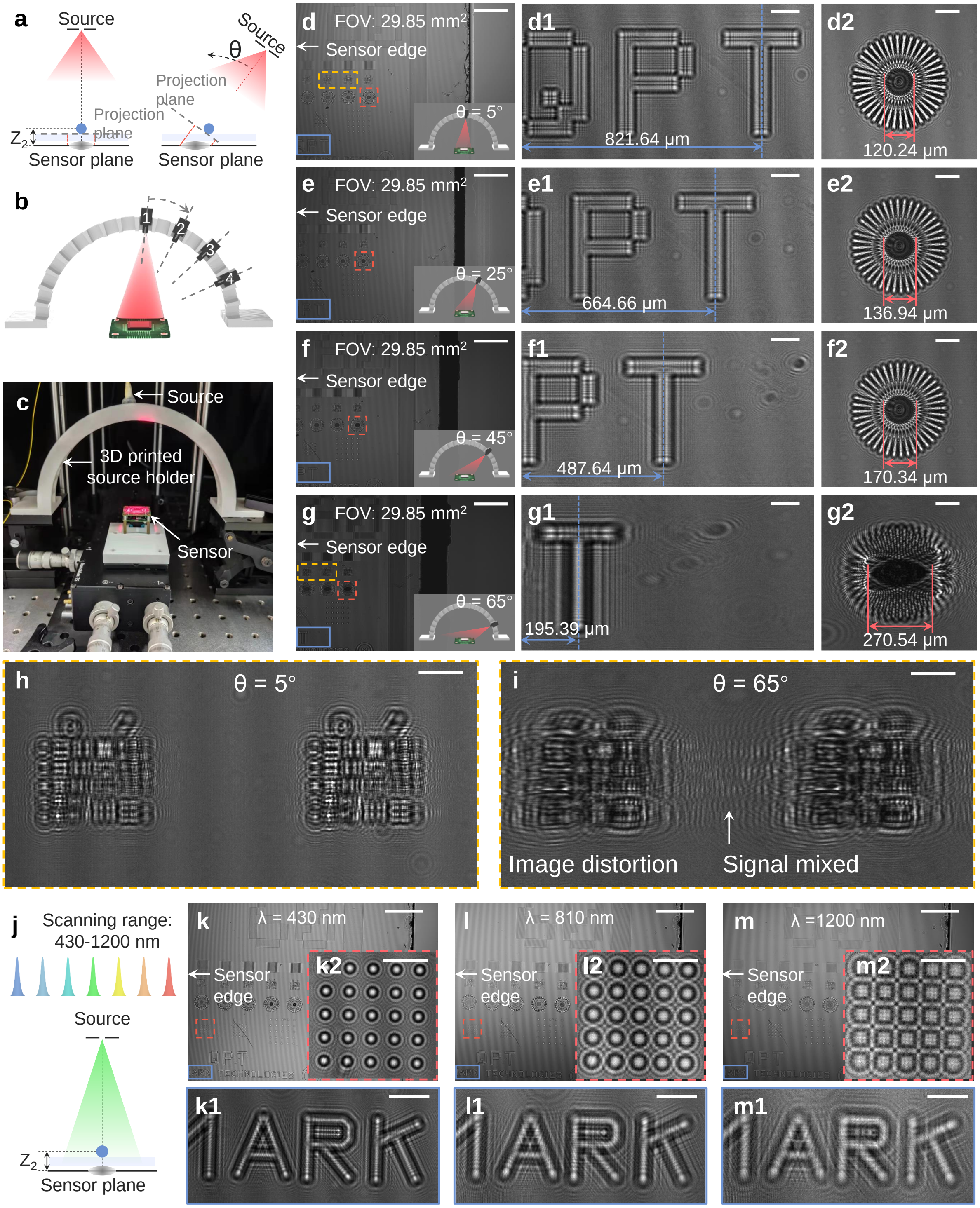

Figure S7. **a** Schematic diagram of holographic imaging under different illumination angles. **b** Conceptual diagram of the experimental design. **c** Photo of the experimental setup. **d-g** Images acquired under 4 different illumination angles, respectively. **d1-g1** Enlarged views of the blue boxes in **d-g**, respectively. **d2-g2** Zoom-ins corresponding to the red dashed boxes in d-g, respectively. **h**, **i** Enlarged displays of the areas marked by the yellow dashed boxes in **d** and **g**, respectively. **j** Schematic diagram of the setup with on-axis illumination. **k-m** Images acquired under 3 different illumination wavelengths, respectively. **k1-m1** Enlarged views of the blue boxes in **k-m**, respectively. **k2-m2** Enlarged views of the red boxes in **k-m**, respectively. Scale bars: 1 mm (Full FOV), 100 μm (Sub-FOV).

and achieve quasi-uniform pixel-super-resolution imaging over the full FOV (Fig. S7j). Figures S7k-m display the diffraction images of the fixed sample captured at λ = 430 nm, 810 nm and 1200 nm, respectively. The blue-boxed areas adjacent to the edge of the sensor are enlarged and displayed in Figs. S7k1-m1. It can be seen that the diffraction pattern of “MARK” expands in place as the wavelength increases, yet the signal features are not lost. The dot matrix (Figs. S7k2-m2) further illustrates that the image maintains its spatial position and the diffraction ring diverges. As summarized in Table S1, we compare the frequency support and reconstruction resolution of LFOCT systems that use either multi-angle or wavelength-scanning illumination schemes. For the LFOCT platform based on angular-scanning illumination, the sensor can capture diffraction patterns under all illumination angles within the central region of the measurable field, thus ensuring an isotropic scattering potential spectrum for the sample. However, near the sensor’s edge, the diffraction patterns are more likely to ‘fly out’ of the measurable area, leading to a reduced number of recorded diffraction patterns and a corresponding anisotropic scattering potential spectrum. Consequently, the imaging resolution diminishes as the distance from the sensor’s edge increases. In contrast, the on-axis wavelength-scanning illumination scheme allows for the recording of diffraction patterns at all wavelengths, maintaining an isotropic scattering potential spectrum and uniform imaging resolution throughout the sensor’s FOV, regardless of the sample’s position.

Finally, it should be noted that the key to high-quality reconstruction is the precise calibration of the imaging system. For experimental systems based on angular scanning, especially those that use a robotic arm to control the scanning of the laser source[17], the repeatability of the experiment depends on the accuracy of the mechanical motion. When conducting multiple experiments, the reciprocal motion introduces cumulative errors that can result in the need for more frequent calibration of the experimental system. In addition, accurately measuring the illumination angle remains challenging, whether using fixed illumination arrays[18,19] or mechanical-scanning source[17]. For the wavelength-scanning-based experimental setup, we only need to use a spectrometer to calibrate the illumination wavelengths. Compared to the measurement of the illumination angle, the calibration process is relatively simple. Since the entire setup is fixed on the optical stage, the system stability depends entirely on the supercontinuum laser and the AOTF, especially the stability of the latter. With the power stability of the AOTF used below 1% (according to the instrument specification), we can safely conclude that our experimental setup exhibits high accuracy and repeatability over extended periods.

Table S1. Comparison between the LFOCT systems based on multi-angle illuminations and only on-axis illumination.

<table>
<tr><td>Illumination scheme</td><td>Multi-angle</td><td>On-axis only</td></tr>
<tr><td>Measurement principle</td><td>Angular scanning</td><td>Wavelength scanning</td></tr>
<tr><td>Frequency support</td><td colspan="2">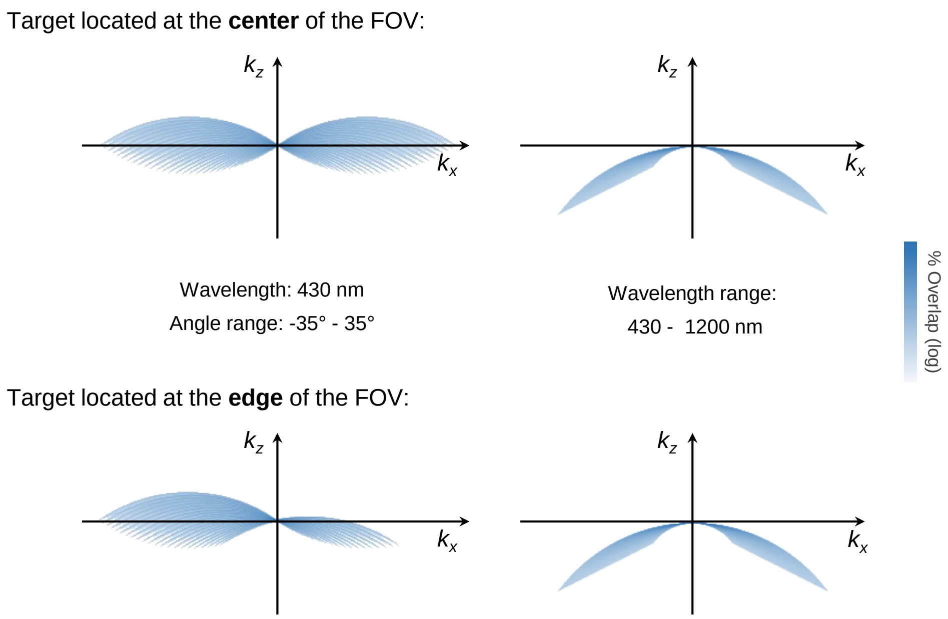
</td></tr>
<tr><td>Full-FOV resolution</td><td>Resolution decreases with distance from the edge</td><td>Quasi-uniform over the full FOV</td></tr>
</table>

Supplementary Note S6. Illustration of wsFPDT method for quasi-uniform pixel-super-resolved tomography over the full FOV

To further illustrate the tomographic capability of the wsFPDT method with quasi-uniform, pixel-super-resolution over the full FOV, the quantitative phase resolution target (PRT) was placed at different positions of the sensor for experimental validation. In the experiment, we placed the PRT directly on the surface of the sensor and calculated its defocus distance to be 414 μm. Then, we select a resolution unit (marked by the black box in Fig. S8a) as the test target for reconstruction, and Fig. S8b shows the details of the test target under the bright-field microscope (10×, 0.45 NA). We placed the test target at the edge and central regions of the sensor FOV (shown in Fig. S8c and d) for the experiments, respectively. Figures S8e and f display the reconstructed 3D RI distributions for the test target located at the edge and center regions of the FOV, respectively. Figures S8e1 and f1 show the RI slices at the focusing plane (z = 0 μm) for the two 3D reconstructed results in Figs. S8e and f, respectively. The elements of Groups 8-9 are zoomed in for comparison (see Figs. S8e2 and f2), and the line profiles in Fig. S8g demonstrates the lateral half-width resolution of 775 nm regardless of whether the test target is located at the edge of the FOV or in the central region. Figures S8e3 and f3 display the $y-z$ cross-sections corresponding to the solid lines in e2 and f2, respectively. The axial line distributions corresponding to Element 3 of Group 9 (Fig. S8h) illustrate the z-axis resolution is 5.43 μm. The RI distributions of the two sets of experimental results (the test target located at the edge and center of the FOV) are essentially the same, supporting the claimed ability of imaging with quasi-uniform pixel super-resolution over the full FOV. Overall, the experimental results based on resolution targets and microspheres are sufficient to demonstrate the feasibility and capability of the proposed wsFPDT to perform non-interferometric accurate 3D RI reconstruction from intensity-only measurements.

However, we must admit that in LFOCT systems, even with on-axis illumination, scattered waves carrying high-frequency information caused by small-sized objects propagate to the sensor plane and they cannot be captured due to the limited FOV. According to the theory of lens-free resolution analysis[16], the reconstruction resolution depends on the size of the reconstruction area we choose and the defocus distance. So, in our LFOCT platform (especially in the edge region), although a lot of information with high-frequency information has gone out from the sensor boundary, the required distance is small for the theoretical super-resolution results we want to achieve, especially compared with that of the oblique illumination and other angular-variable methods. For example, in our system, the defocus distance Z_2 = 420 μm is known, and to achieve a half-width resolution of 0.775 μm, the side length of the minimum reconstruction sub-FOV is 242.6 μm. That is, to ensure the accurate capture of essential high-frequency information, it is recommended to position the center of the targeted area at a minimum distance of 121.3 μm away from the sensor edge.

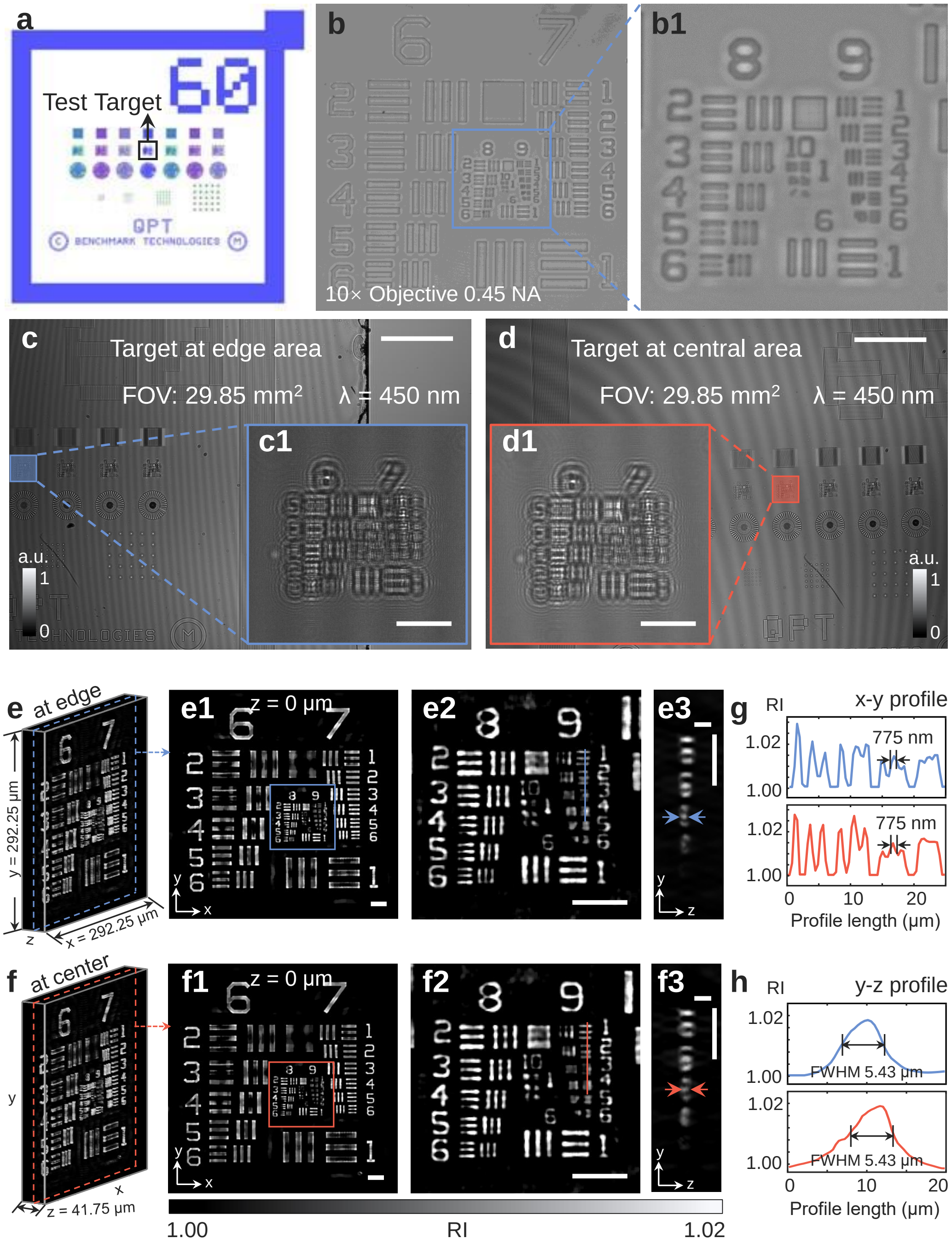

Figure S8. **a** Illustration diagram of the full-view pure phase resolution target. **b** Imaging results of the test target under a 10× objective. **b1** Magnified image of the blue boxed area in b. **c**, **d** Full-FOV diffraction patterns acquired directly by the sensor when the test target is located at the edge and central regions of the sensor's FOV. **c1** (**d1**) Magnified image of the boxed area in c (d). **e**, **f** 3D RI distributions of the test target in c and d reconstructed using the wsFPDT method, respectively. **e1**, **f1** The $x-y$ RI cross-sections at $z = 0$ μm (focus plane) in e and f, respectively. **e2**, **f2** Enlargements of the selected regions including Groups 8-9 in e1 and f1, respectively. **e3**, **f3** The $y-z$ cross-sections corresponding to the solid lines in e2 and f2, respectively. **g** The line distributions corresponding to the solid lines through Elements 1-4 of Group 9 in e2 and f2. **h** The axial line distributions corresponding to Element 3 of Group 9 in e3 and f3. Scale bars: 1 mm (Full FOV), 20 μm (Sub-FOV).

Supplementary Note S7. Comparison of the wsFPDT method with the swept-source optical coherence tomography (SS-OCT) technique

The wsFPDT method proposed in this work is conceptually similar to the swept-source optical coherence tomography (SS-OCT) technique[20–22], which also adopts a wavelength-scanning illumination scheme to probe 3D information of biological samples. Instead of using full-field detection based on a 2D array detector, the SS-OCT technique is commonly implemented in a point-by-point scanning manner with a high-speed point photodetector to measure the interference signal of the backscattered object beam with the reference beam. The longitudinal single-point depth scan (i.e., A-scan, a term borrowed from ultrasound imaging) is achieved by scanning from short to long with fast tuning wavelength in a single scan cycle. And longitudinal cross-sectional scan images (i.e., B-scan) are achieved by moving the probe laterally, and multiple B-scan data are synthesized to build up a 3D volumetric image. Despite the conceptual similarity, there are several fundamental differences between these two methods, as compared in Table S2.

Table S2. Comparison between the SS-OCT and wsFPDT methods.

Methods	SS-OCT	wsFPDT
Detection principle	Interferometric	Non-interferometric
Measurement principle	Point-by-point scanning	Full field (w/o moving devices)
Detected signal	Backward scattering (dark field)	Forward scattering (bright field)
Spectral coverage	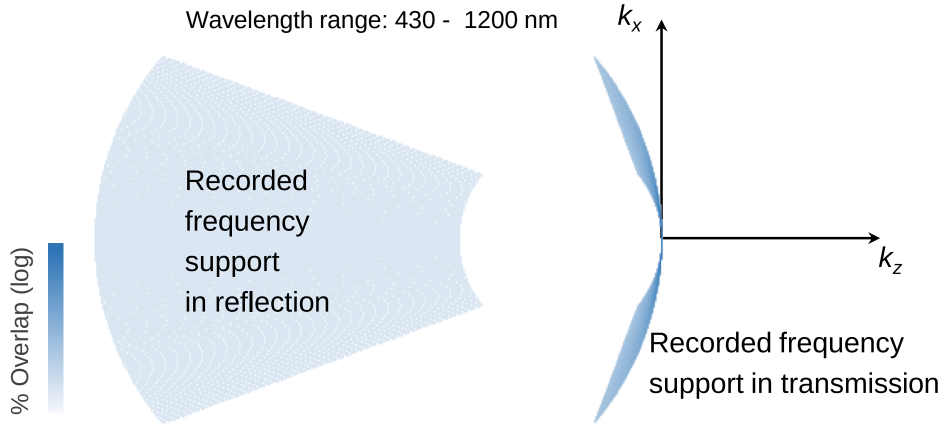	
Suited sample	Strongly scattering (tissues)	Weakly scattering (cells)
Result interpretation	Obscured	Quantitative RI

As summarized in Table S2, the distinct advantages of the wsFPDT over the SS-OCT include its

non-interferometric detection principle and full-field measurement characteristic, allowing motion-free volumetric imaging with a relatively simple setup. In terms of the detected signal, the SS-OCT measures the backward scattering field, providing a much wider spectral coverage, especially along the axial direction. Since there is no overlap between the spectrum corresponding to each wavelength, SS-OCT employs interferometry to measure phase[23,24]. But the introduction of interferometric optical paths increases the complexity of the configuration. In contrast, the wsFPDT method measures the forward scattering field, and the Ewald sphere is tangentially overlapping at the k-space origin, resulting in limited spectral coverage. However, the inherent sufficient overlapping between the spectra can be used for phase recovery. Moreover, it can be found that there is a complete absence of low-frequency information in an SS-OCT spectrum. In other words, the relationship between the phase, thickness, and RI of the object is completely obscured in the SS-OCT measurement, and the signal is generally a dark-field speckle pattern. On the other hand, the wsFPDT method can recover the quantitative RI distribution of the sample containing low-frequency information by measuring the bright field transmitted signal. Regarding the measurable sample type, although the reflection-based geometrical configuration allows the SS-OCT method to achieve high axial resolution, it is only suitable for strongly scattering samples that can produce strong backscattered signals, such as biological tissues. Whereas most biological cells are transparent and weakly scattering such that they produce almost no backscattering signal when being illuminated with a plane wave. In such cases, the wsFPDT is more suitable to recover their morphology and quantitative RI distributions, despite its relatively low axial resolution.

References

1. Hofmann, P. *Solid state physics: an introduction* (John Wiley & Sons, 2022).
2. Wolf, E. Three-dimensional structure determination of semi-transparent objects from holographic data. *Opt. Commun.* **1**, 153–156 (1969).
3. Sung, Y. *et al.* Optical diffraction tomography for high resolution live cell imaging. *Opt. Express* **17**, 266–277 (2009).
4. Kak, A. C. & Slaney, M. *Principles of computerized tomographic imaging* (SIAM, 2001).
5. Devaney, A. Inverse-scattering theory within the rytov approximation. *Opt. Lett.* **6**, 374–376 (1981).
6. Pech-Pacheco, J. L., Cristóbal, G., Chamorro-Martinez, J. & Fernández-Valdivia, J. Diatom autofocusing in brightfield microscopy: a comparative study. In *Proceedings 15th International Conference on Pattern Recognition. ICPR-2000*, vol. 3, 314–317 (IEEE, 2000).
7. Mudanyali, O., Oztoprak, C., Tseng, D., Erlinger, A. & Ozcan, A. Detection of waterborne parasites using field-portable and cost-effective lensfree microscopy. *Lab on a Chip* **10**, 2419–2423 (2010).
8. Zhang, J., Sun, J., Chen, Q., Li, J. & Zuo, C. Adaptive pixel-super-resolved lensfree in-line digital holography for wide-field on-chip microscopy. *Sci. Reports* **7**, 11777 (2017).
9. Zuo, C., Sun, J. & Chen, Q. Adaptive step-size strategy for noise-robust fourier ptychographic microscopy. *Opt. Express* **24**, 20724–20744 (2016).
10. Lim, J. *et al.* Comparative study of iterative reconstruction algorithms for missing cone problems in optical diffraction tomography. *Opt. Express* **23**, 16933–16948 (2015).
11. Kim, K. *et al.* Optical diffraction tomography techniques for the study of cell pathophysiology. *J. Biomed. Photonics & Eng.* **2**, 020201–1 (2016).
12. Park, S., Lee, L. E., Kim, H., Kim, J. E. & Lee, S. Detection of intracellular monosodium urate crystals in gout synovial fluid using optical diffraction tomography. *Sci. Reports* **11** (2021).
13. Kim, K., Kim, K. S., Park, H., Ye, J. C. & Park, Y. Real-time visualization of 3-d dynamic microscopic objects using optical diffraction tomography. *Opt. Express* **21**, 32269–32278 (2013).
14. Horstmeyer, R., Chung, J., Ou, X., Zheng, G. & Yang, C. Diffraction tomography with fourier ptychography. *Opt.* **3**, 827–835 (2016).
15. Ozcan, A. & McLeod, E. Lensless imaging and sensing. *Annu. review biomedical engineering* **18**, 77–102 (2016).
16. Zhang, J., Sun, J., Chen, Q. & Zuo, C. Resolution analysis in a lens-free on-chip digital holographic microscope. *IEEE Transactions on Comput. Imaging* **6**, 697–710 (2020).
17. Isikman, S. O. *et al.* Lens-free optical tomographic microscope with a large imaging volume on a chip. *Proc. Natl. Acad. Sci.* **108**, 7296–7301 (2011).

18. Zuo, C., Sun, J., Zhang, J., Hu, Y. & Chen, Q. Lensless phase microscopy and diffraction tomography with multi-angle and multi-wavelength illuminations using a LED matrix. *Opt. Express* **23**, 14314–14328 (2015).

19. Luo, Z. *et al.* Fast compressive lens-free tomography for 3d biological cell culture imaging. *Opt. Express* **28**, 26935–26952 (2020).

20. Choma, M. A., Sarunic, M. V., Yang, C. & Izatt, J. A. Sensitivity advantage of swept source and fourier domain optical coherence tomography. *Opt. Express* **11**, 2183–2189 (2003).

21. Lexer, F., Hitzenberger, C. K., Fercher, A. & Kulhavy, M. Wavelength-tuning interferometry of intraocular distances. *Appl. Opt.* **36**, 6548–6553 (1997).

22. Chinn, S., Swanson, E. & Fujimoto, J. Optical coherence tomography using a frequency-tunable optical source. *Opt. Lett.* **22**, 340–342 (1997).

23. Huang, D. *et al.* Optical coherence tomography. *Sci.* **254**, 1178–1181 (1991).

24. Ralston, T. S., Marks, D. L., Carney, P. S. & Boppart, S. A. Interferometric synthetic aperture microscopy. *Nat. Phys.* **3**, 129–134 (2007).
